# Advancements in Asymmetric Supercapacitors: From Historical Milestones to Challenges and Future Directions

**DOI:** 10.1002/advs.202403172

**Published:** 2024-07-09

**Authors:** Shrikant Vaiju Sadavar, Seul‐Yi Lee, Soo‐Jin Park

**Affiliations:** ^1^ Department of Chemistry Inha University 100 Inharo Incheon 22212 Republic of Korea

**Keywords:** asymmetric supercapacitors, basic principles, categorization, electrode materials, industrial applications

## Abstract

Numerous challenges, like the uninterrupted supply of electricity, stable and reliable power, and energy storage during non‐operational hours, arise across various industries due to the absence of advanced energy storage technologies. With the continual technological advancements in portable electronics, green energy, and transportation, there are inherent limitations in their innovative production. Thus, ongoing research is focused on pursuing sustainable energy storage technologies. An emerging solution lies in the development of asymmetric supercapacitors (ASCs), which offer the potential to extend their operational voltage limit beyond the thermodynamic breakdown voltage range of electrolytes. This is achieved by employing two distinct electrode materials, presenting an effective solution to the energy storage limitations faced by ASCs. The current review concentrates on the progression of working materials to develop authentic pseudocapacitive energy storage systems (ESS). Also, evaluates their ability to exceed energy storage constraints. It provides insights into fundamental energy storage mechanisms, performance evaluation methodologies, and recent advancements in electrode material strategies. The review approaches developing high‐performance electrode materials and achieving efficient ASC types. It delves into critical aspects for enhancing the energy density of ASCs, presenting debates and prospects, thereby offering a comprehensive understanding and design principles for next‐generation ASCs in diverse applications.

## Graphical abstract

Exploring asymmetric supercapacitors (ASCs) presents a breakthrough in overcoming energy storage constraints through innovative design of distinct electrode materials. This review delves into recent progress in materials, storage mechanisms, performance assessments, and strategies to boost energy density. Such advancements are crucial for industrial applications and the advancement of energy storage systems (ESS).

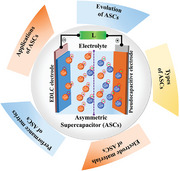

## 1 Introduction

The global surge in energy demand propels the development of high‐power alternative sources. Devices such as smartphones and tablets, interconnected in our daily lives, necessitate robust energy storage solutions for continuous operation. A decade ago, electrochemical capacitors (ECs) sufficed for rapid energy needs; however, today's requirements for versatile, high‐power solutions have intensified. Predominant alternative sources like batteries, fuel cells, and supercapacitors, all operating on electrochemical principles, play a pivotal role in consumer electronics, including cars, phones, laptops, and cameras. These technologies are integral to navigating the evolving energy landscape, fulfilling increasing power needs, and ensuring uninterrupted operation of daily‐use devices.^[^ ^1^, ^2^ ^]^ Supercapacitors, in particular, find applications in heavy‐duty vehicles, renewable energy systems, and electric vehicles’ regenerative braking energy.

Despite their higher energy density (≈5 Wh kg^−1^) compared to traditional capacitors, supercapacitors lag behind batteries (≈200 Wh kg^−1^) and fuel cells (≈350 Wh kg^−1^) in this aspect, limiting their widespread use. Consequently, ongoing research efforts aim to enhance supercapacitors energy density to rival batteries while maintaining their power density and cycling stability.^[^ ^3^ ^]^ Terminologies in supercapacitor technology, including asymmetric, pseudocapacitors, and hybrid supercapacitors, are widely recognized for describing them. An asymmetric capacitor (ASC) employs electrodes of differing capacitances, separated by an electrolyte. In this setup, the larger electrode, made of a material with superior specific capacitance, has a higher absolute capacitance than the smaller one. Interestingly, the physical size of the larger electrode can be comparable to, or even smaller than, the smaller capacitance electrode.^[^ ^4^, ^5^ ^]^

ASCs enhance the charge and discharge processes by offering distinct potential windows for each electrode, thereby increasing the device's operational voltage. Contrary to aqueous‐based symmetric systems limited to ≈1.2 V, ASCs can exceed 2.0 V in operating voltage, significantly boosting their energy density. Herein, to achieve a higher potential window in ASCs by applying stable electrode materials and advanced electrolyte preparations like water‐in‐salt electrolytes which significantly surge the stability window of water. Also, the solid‐state electrolytes defeat water electrolysis. Steady electrode materials and surface functionalization boost the tolerance of voltage. Moreover, the potential window of an electrolyte is described by its oxidative and reductive potential boundaries. Electrode redox potentials must be inside the potential window lest a stable solid electrolyte boundary is established.^[^ ^6^ ^]^ Aqueous electrolyte characteristics, including solvents and anion carriers, influence the thermodynamics of anion‐storing capacitors. Numerous anion carriers (e.g., OH^−^, Cl^−^, F^−^, ClO_4_^−^) in several solvents influenced the potential window. In aqueous electrolytes, the thermodynamic potential window of water is 1.23 V, with potential moves due to pH alterations, while the potential window rests uniformly. Enriched electrolyte‐electrode interfaces and shielding additives further boost system stability and energy density.^[^ ^7^ ^]^ This advancement was pivotal in establishing ASCs as commercially viable energy storage devices. In 1978, Nippon Electric Corp. (NEC) made a significant stride by commercializing the first electrochemical capacitor, licensed from SOHIO, and branded it as a supercapacitor. NEC's pioneering role in introducing this technology to the market primarily targeted backup power for clock chips and complementary metal−oxide−semiconductor memories within electronic devices.^[^ ^3^, ^8^ ^]^ Since then, supercapacitor research has advanced considerably, driven by introducing novel physicochemical characterization techniques and developing various nanostructured materials.^[^ ^9^, ^10^ ^]^ Presently, commercially available supercapacitors still trail battery systems in energy density, spurring research efforts to elevate this critical parameter. The growing demand for higher energy density aligns with the evolving needs of next‐generation electronic devices, underscoring the urgent necessity to further enhance supercapacitors energy density.^[^ ^11^, ^12^ ^]^

Furthermore, the demand for sustainable energy solutions intensifies as the world grapples with fossil fuel depletion and heightened environmental concerns. While solar and wind energy offer potential, an efficient energy storage systems (ESS) remains a paramount challenge. Lithium‐ion batteries, predominantly powering consumer electronics, face issues like overheating and safety. Supercapacitors, emerging as an alternative, pave the way for advancing ASCs. These configurations, improving voltage during charge and discharge cycles, address the energy density limitation and find applications in high‐power settings.^[^ ^13^, ^14^ ^]^ This review delves deeply into ASC systems, their design, mechanisms, materials, and performance evaluation, offering critical insights into recent advancements and future research directions.

## 2 Evolution, Basic Principles, and Energy Storage Mechanism of ASC

### 2.1 Evolution

The history of supercapacitors traces back to the 18^th^ century with the invention of the Leyden jar. This early capacitor, developed in 1745, marked the beginning of our understanding of charge storage. Over the ensuing years, the concept and technology of storing static electricity at the interface of a solid electrode and a liquid electrolyte evolved significantly.^[^ ^15^ ^]^ In the 19^th^ century, researchers like von Helmholtz explored the electrical charge‐storage mechanism, laying the groundwork for the modern theory of electric double‐layer (EDL) capacitance. However, it was not until 1954 that the first electrochemical capacitor patent emerged, outlining an energy storage device featuring porous carbon electrodes.^[^ ^16^, ^17^ ^]^

The momentum continued in 1978 when NEC commercialized the first non‐aqueous electrolyte‐based electrochemical capacitor. This breakthrough initiated its applications in backup power for electronic devices. In the 1980s, the discovery of pseudocapacitance, which includes Faradaic processes, spurred the development of high‐performance supercapacitors. Nevertheless, the high cost of materials limited some of these applications to military use. In 1989, the U.S. Department of Energy studied high‐energy‐density supercapacitors, leading to collaborations with companies such as Maxwell Technologies.^[^ ^18^, ^19^ ^]^

**Figure 1:**
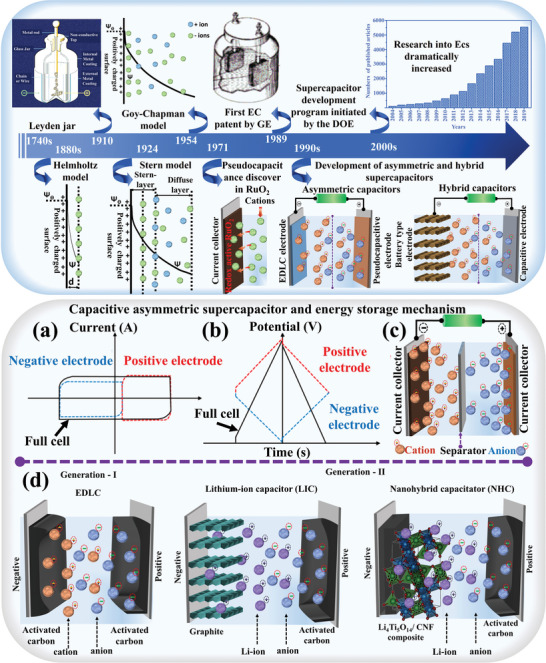
(Top panel) Historical evolution of supercapacitor. The schematics of the Helmholtz, Gouy−Chapman, Stern models, adapted with permission.^[^ ^24^ ^]^ Copyright 2009, Royal Society of Chemistry, and publication graph adapted with permission.^[^ ^28^ ^]^ Copyright 2023, Elsevier.^[^ ^24^, ^25^ ^]^ (Bottom panel) Schematic illustration of a) CV curve, b) GCD curve, adapted with permission.^[^ ^3^ ^]^ Copyright 2018, American Chemical Society.^[^ ^27^ ^]^ c) energy storage mechanism of capacitive ASCs adapted with permission.^[^ ^28^ ^]^ Copyright 2023, Elsevier and d) schematic representation of charge storage mechanism in first and second generation ASCs, adapted with permission.^[^ ^29^ ^]^ Copyright 2021, Elsevier.^[^ ^26^, ^27^, ^28^, ^29^, ^30^ ^]^

The subsequent years saw the emergence of various types of supercapacitors, each distinguished by its unique characteristics and applications. Since 2000, supercapacitor research has accelerated, driven by the growing demand for high‐power, reliable, and safe energy storage devices. The evolution of the supercapacitor is schematically represented in **Figure** **1** (Top panel). The advancement of nanoscience and sophisticated characterization techniques continues to uncover new phenomena, prompting further investigation into the charge‐storage mechanisms of EDLCs and pseudocapacitances.^[^ ^17^, ^19^ ^]^

Consequently, ASCs were established to resolve the limitations of symmetric supercapacitors by merging electrodes with broader potential windows and high capacitance. Inventions in active materials, comprising advanced carbons, conducting polymers, and transition metal oxides have enhanced performance. Accordingly, developed electrolytes such as ionic liquids and water‐in‐salt are used in ASCs that can enable the extension of voltage windows. Moreover, engineering developments in electrode matching, hybrid arrangements, and nano‐structuring materials further improved ASCs. Nowadays, evolution comprises flexible designs and solid‐state devices. Future guidelines focus on participating ASCs with hybrid ESS, advanced displaying techniques, and smart grids, dazzling a multidisciplinary tactic to enlighten performance and escalating commercial‐level applications.^[^ ^20^, ^21^, ^22^ ^]^

### 2.2 Basic Principles

ASCs encompass a wide theoretical range by incorporating two distinct electrode materials. This design facilitates the creation of hybrid devices, which can feature varying ratios of redox‐active sites or employ different charge‐storage mechanisms. In addition, the principle of selecting ASC electrodes is illustrated with the work function mechanism (Figure S1, Supporting Information).^[^ ^23^ ^]^ The work function can vary nearby even on the same electrode which is influenced by both electrochemical potentials and surface. Surface potential is hard to control because it depends on lots of factors such as surface irregularity, reform, crystallographic orientation, and adsorbed external entities. Highly pure materials and careful preparation can diminish it but governing the surface potential is still challenging.

According to an increase in the potential window of supercapacitors, it is better to know the redox potential of active or electrode material. Based on the first law of thermodynamics by the Born‐Haber cycle, it states the change in overall energy during electrode reaction. Consider a half‐cell reaction of electrode material “m” in an aqueous solution in which ferrous ions (Fe^2+^) occur after the reduction of ferric ions (Fe^3+^). Therefore, a change in Gibbs free energy is represented in Equation (1).

(1) $$\Delta G=-G_{s,\;Fe^{3+}}+\phi_{m}+\left(-I_{Fe^{2+}}\right)+G_{s,\;Fe^{2+}}$$

where G_s_, I, and ϕ_m_ are the Gibbs energy of hydration for Fe^2+^ and Fe^3+^ ions (values are large negative), the ionization energy of Fe^2+^ transforming to Fe^3+^ (in the gas phase), and the electronic work function of electrode material's, respectively. In ASCs with unalike electrode materials, the potential window is provided as follows.

(2) $$E_{\mathrm{cell}}=E_{0}+\Delta E_{1}+\Delta E_{2}$$

(3) $$E_{\mathrm{cell}}=\frac{1}{F}.\left(\omega^{\beta }-\omega^{\alpha }\right).N_{A}+\Delta E_{1}+\Delta E_{2}$$

(4) $$E_{\mathrm{cell}}=\left[\left(\frac{1}{F}.\omega^{\beta }.N_{A}\right)+\Delta E_{1}\right]+\left[\left(\frac{1}{F}.\omega^{\alpha }.N_{A}\right)+\Delta E_{2}\right]$$

(5) $$\begin{array}{ccc} E_{\mathrm{cell}} & = & \left[\mathrm{Work}\;\mathrm{function}\;\left(\mathrm{electrode}\;1\right)\right]\\ & & +\;\left[\mathrm{Work}\;\mathrm{function}\;\left(\mathrm{electrode}\;2\right)\right] \end{array}$$

where ω^β^, ω^α^, N_A_, E_0_, (ΔE_1_ and ΔE_2_), and F are the electrochemical potentials of the positive electrode, the electrochemical potentials of the negative electrode, Avogadro's constant, the standard electrode potential, the surface dipole potentials, and the Faraday constant, respectively.^[^ ^19^ ^]^ The working voltage is determined by the electrolyte dissociation energy, with a maximum of 1.23 V in an aqueous electrolyte. An ASC has different surface potentials and various work functions due to the comprises of uneven electrode materials. Consequently, the potential window is the difference between the work functions of the two electrodes. Thus, this must be studied considering i) work function alterations due to the solvated electrolyte ions adsorption, ii) energy levels of an electrolyte, and iii) effects of oxygen and hydrogen evolution overpotentials on the electrodes. In contrast, the identical material is employed for both electrodes in a symmetric supercapacitor. Therefore, it shows zero additional potential window due to ω^β^ = ω^α^ and ΔE_1_ = ‐ΔE_2_.^[^ ^23^ ^]^ Notably, hybrids with Faradaic electrodes, such as RuO_2_, MnO_2_, NiO, Ni(OH)_2,_ or Co_3_O_4_, in conjunction with carbon electrodes, are typical examples in this category. In contrast, capacitive ASCs are characterized by two capacitive‐type electrodes, yielding an ideal rectangular CV curve and a triangular GCD curve for the device. These typical CV and GCD curves of capacitive ASCs are schematically depicted in Figure 1a,b (Bottom panel).^[^ ^17^, ^24^, ^26^, ^27^ ^]^

### 2.3 Energy Storage Mechanism

The energy storage mechanism in ASCs involves employing distinct electrode materials for the negative and positive electrodes. This approach leads to a synergistic combination of EDLCs and pseudocapacitance during the charge and discharge processes.^[^ ^31^ ^]^ The ASCs integrate the rapid charge/discharge characteristic of EDLC at the negative electrode with the enhanced energy storage capability of pseudocapacitance at the positive electrode. This combination results in a highly efficient and high‐performance ESS. For instance, Subramani et al. developed ASCs utilizing transition metal‐based compounds (TMC) with an alkali/polyvinyl alcohol (KOH/PVA) gel electrolyte. In their design, commercially available activated carbon was used for the negative electrode, while CoS derived from cobalt hexacyanoferrate served as the positive electrode material.^[^ ^32^ ^]^

Figure 1c (Bottom panel) illustrates the schematic and energy storage mechanism of ASCs. During the charging and discharging phases, the gel electrolyte's K^+^ and OH^−^ migrate toward the respective negative and positive electrodes. K^+^ undergoes adsorption/desorption at the negative electrode, forming an electrical double layer. Conversely, the positive electrode undergoes a reversible faradaic process involving the Co^2+^/Co^3+^ and Co^3+^/Co^2+^ redox couples in the presence of OH^−^. Consequently, the negative electrode in the ASCs functions primarily as EDLC, while the positive electrode facilitates energy storage through pseudocapacitance via a reversible faradaic process.^[^ ^26^, ^31^ ^]^ Moreover, a schematic representation of the charge storage mechanism in first and second‐generation ASCs shown in Figure 1d (Bottom panel).

## 3 Types of ASCs

**Figure 2:**
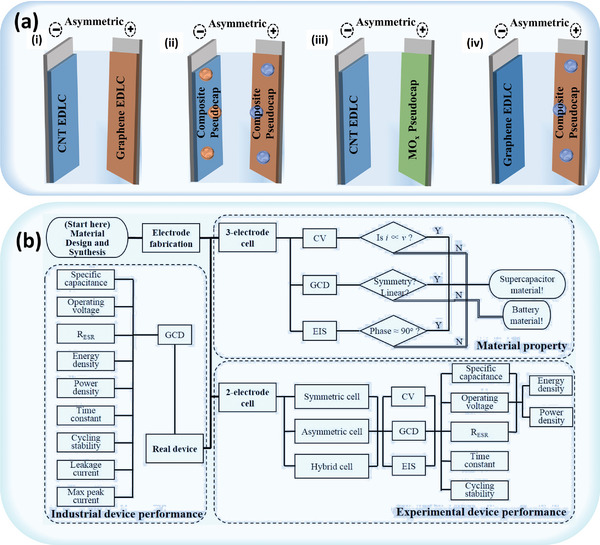
a) Types of ASCs, adapted with permission.^[^ ^38^ ^]^ Copyright 2022, multidisciplinary digital publishing institute and b) flowchart for the characterization of electrode materials and devices, adapted with permission.^[^ ^11^ ^]^ Copyright 2018, Elsevier.

Supercapacitors are tailored based on the composition of their electrodes. In symmetric supercapacitors, both the negative and positive electrodes consist of the same materials, typically carbon‐based. Supercapacitors rapidly store and deliver high‐power electricity. They comprise two electrodes separated by an electrolyte in which materials need a high surface area to accumulate plentiful charges via physical absorption. In lithium(Li)‐ion battery comprises an anode, a cathode, an electrolyte, and a separator. During charging, Li^+^ ions move from the cathode to the anode, harmonizing electron drift in the exterior circuit. Moreover, the comparison of supercapacitors with Li‐ion batteries based on functions and characteristics is provided in Table S1 (Supporting Information). Also, the advantages and disadvantages of the supercapacitor, Li‐ion battery, Na‐ion battery, and solid‐state battery are provided in Table S2 (Supporting Information).^[^ ^33^, ^34^, ^35^ ^]^ In contrast, ASCs feature a wide range of electrode material combinations, as depicted in **Figure** **2**a. Furthermore, for greater convenience, ASCs can be systematically categorized according to their electrochemical components.^[^ ^24^ ^]^

### 3.1 Categorization of ASCs Based on Their Electrochemical Components (Capacitive and Pseudocapacitive)

In this section, ASCs exclusively use pseudocapacitive or capacitive electrodes. These energy storage devices exhibit reversible operation across stable potential ranges and can employ EDLC, pseudocapacitance, and battery‐type mechanisms. Enhancing operating voltage and capacitances significantly improves the energy and power density of these devices.^[^ ^2^, ^36^, ^37^ ^]^

### 3.2 Capacitive ASCs

In this context, capacitive ASCs solely utilize capacitive or pseudocapacitive electrodes. Enhancing operating voltage and capacitances can substantially improve energy and power density. In EDLCs, from a capacitive perspective, carbon‐based electrodes, electrolytes, and a separator are featured, storing charges electrostatically or through a non‐Faradaic method. Common carbon electrode materials for supercapacitors, such as activated carbon, carbon aerogel, graphene, graphite, and carbon nanotubes (CNTs), exhibit capacitive behavior, despite their lower electrical conductivity compared to metals. Supercapacitor electrodes, crucial components in the capacitive region, are thin sheets connected to a conductive current collector. They require qualities such as environmental friendliness, cost‐effectiveness, admirable conductivity, low corrosion resistance, and durable chemical stability.^[^ ^31^, ^37^, ^39^ ^]^ Moreover, capacitive material is specifically configured, characterized, and identified, as depicted by the flowchart in Figure 2b. In the material property zone illustrated, pseudocapacitive materials can fulfill the required electrochemical features.^[^ ^11^ ^]^

Activated carbon is widely used, particularly in consolidated amorphous carbon (CAC) form. Activated carbon fibers contribute to its capacitive nature. Carbide‐derived carbon (CDC) offers tunability and nanoporosity, and random porous carbons are preferred for their capacitive advantages. Graphene, arranged hexagonally, is extensively utilized in nanocomposite paper form, aligning with capacitive principles. MnO_2_ and RuO_2_, employed in pseudocapacitors, exhibit both capacitive and Faradaic behavior, further emphasizing the capacitive nature of these ESS.^[^ ^39^ ^]^ However, the key challenges in achieving high energy and power densities in capacitive ASCs revolve around carefully selecting suitable electrode materials and the thoughtful design of device configurations.^[^ ^5^, ^31^, ^37^ ^]^

**Figure 3:**
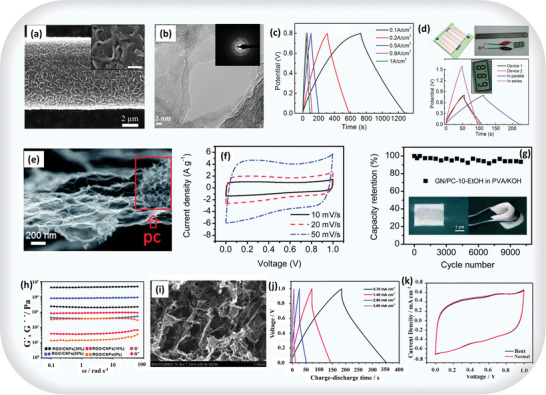
Capacitive activity in ASCs based on carbon materials: a) SEM image of PPy wrapping on the MnO_2_‐CF, scale bars of the insets in 1 µm, b) HRTEM image and the inset SAED pattern of MnO_2_ nanoflakes showing the polycrystalline nature of MnO_2_, c) GCD curves of the device under different current densities, and d) schematic of the three SCs connected in series; optical image of the three SCs in series drove an LCD; GCD curves of single SC and two SCs connected in series or in parallel, adapted with permission.^[^ ^40^ ^]^ Copyright 2013, Springer Nature, e) typical FESEM images of GN/PC‐10‐EtOH, f) CV curves of GN/PC‐10‐EtOH in PVA/KOH gel electrolyte, g) cycling stability of GN/PC‐10‐EtOH in PVA/KOH gel electrolyte, adapted with permission.^[^ ^41^ ^]^ Copyright 2012, Royal Society of Chemistry, h) dynamic rheological behavior of the CNF‐based hydrogels, i) RGO–CNFs (20%) hybrid aerogel, j) typical galvanostatic charge–discharge curves of the ASCs at different current densities, and k) comparison of CV curves at 5 mV s^−1^ for ASCs tested in normal and bent states, adapted with permission.^[^ ^42^ ^]^ Copyright 2012, Royal Society of Chemistry.

Moreover, multiple reports confirm success in achieving high capacitance with heteroatom‐doped carbon materials in alkaline electrolytes. For example, Jiayou Tao et al. optimized electrochemical performance using MnO_2_‐based electrodes by electro‐polymerizing polypyrrole (PPy) and wrapping it onto MnO_2_ nanoflakes (**Figure** **3**a). This process created an organic‐inorganic composite coating on carbon fiber (CF) with ε‐MnO_2_, forming a high‐performance supercapacitor (SC). The polycrystalline MnO_2_, confirmed by high‐resolution transmission electron microscopy (HRTEM) and SAED patterns, exhibited robust capacitance performance across various current densities (Figure 3b and inset). The PPy‐MnO_2_‐CF, ≈9.5 mm in diameter, achieved remarkable volume capacitance: 69.3 F cm^−3^ at 0.1 A cm^−2^, surpassing literature values (Figure 3c). A schematic of three supercapacitors connected in series demonstrated predictable capacitance rules, offering versatility in practical applications. Test results in Figure 3d showed that devices connected in series had an overall capacitance of 0.11 mF, and in parallel, it reached 0.48 mF, aligning with fundamental capacitor connection principles. An application example in Figure 3d (top) showcased three series‐connected SCs effectively powering a commercial LCD when fully charged, highlighting the practicality and versatility of such interconnected SCs.^[^ ^40^ ^]^

Likewise, Hong‐Fei Ju et al. reported that the addition of polycarbonate (PC) prevents graphene restacking in hydrothermal processing, resulting in looser GN/PC aerogel structures (Figure 3e). The GN/PC‐10‐E_t_OH electrode in an all‐solid‐state supercapacitor exhibits ideal capacitive behavior (Figure 3f) at a high scan rate (50 mV s^−1^), maintaining remarkable cycling stability (less than 10% loss after 10000 cycles) in a PVA/KOH electrolyte (Figure 3g). Compared to other materials, GN/PC promises long‐lasting energy storage. Additionally, GN/PC aerogels form flexible supercapacitors with good mechanical flexibility (inset of Figure 3g). Notably, mechanical bending does not affect their electrochemical performance. In summary, the integration of PC renders the GN/PC aerogel an exemplary choice for flexible all‐solid‐state supercapacitor electrodes due to its unique structure, cycling stability, and mechanical flexibility.^[^ ^41^ ^]^

Similarly, Kezheng Gao et al. proposed through a rheological study, as shown in Figure 3h, that the CNF–RGO hybrid hydrogels exhibit a predominant elastic response, indicative of a permanent network. Graphene oxide values are lower than those of pure RGO but higher than those of pure CNFs, implying an effective reduction of *π*–*π* stacking interactions by CNFs. This reduction leads to uniform *π*–*π* stacking interactions, resulting in a 3D porous structure with sub‐micrometer micrometer‐scale pores (Figure 3i). Traditionally, dense *π*–*π* stacking in 3D graphene networks limits electrochemical performance. In contrast, the rough pore walls of the CNF–RGO (20%) hybrid aerogel demonstrate interconnected RGO with individual graphene nanosheet characteristics, favoring electron transport. Therefore, choosing CNF–RGO (20%) as a supercapacitor electrode material is justified. Galvanostatic charge–discharge curves, depicted in Figure 3j, demonstrate excellent capacitive performance (203 F g^−1^ at 0.7 mA cm^−2^). Electrochemical stability during bending is crucial for flexible supercapacitors, with CV curves in Figure 3k, exhibiting minimal changes in the bent state, affirming robust performance even after 100 bending cycles.^[^ ^42^ ^]^ Accordingly, recently reported capacitive ASCs with cell configuration and electrochemical performance are summarized in Table S3 (Supporting Information).^[^ ^43^, ^44^, ^45^, ^46^, ^47^, ^48^, ^49^, ^50^, ^51^, ^52^ ^]^

### 3.3 Hybrid Capacitor

A hybrid capacitor combines double layer and pseudocapacitance with polarizable (carbon) and non‐polarizable (metal or polymer) electrodes. Its operation involves both Faradaic and non‐Faradaic processes. Faradaic charge storage relies on electrochemical redox reactions, adhering to Faraday's law. In systems like batteries, the electrode‐electrolyte interface resembles that of a supercapacitor, raising questions about capacitance. The electrical double‐layer capacitance (CDL) is present in both capacitive and Faradaic interfaces but is often negligible in Faradaic energy storage devices. CDL, typically ranging from 10 to 40 µF cm^−2^ in diffusion‐limited batteries, is described by models such as Helmholtz, Gouy‐Chapman, and Stern. The structure of the electrical double layer has been extensively analyzed in other studies.^[^ ^53^, ^54^ ^]^

**Figure 4:**
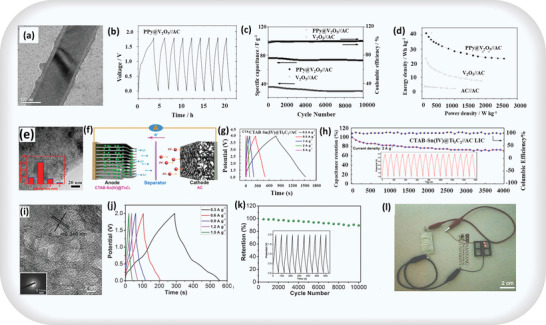
Capacitive activity in Faradaic ASCs: a) TEM image of core–shell‐structured PPy@V_2_O_5_, b) charge/discharge curves of the assembled asymmetric PPy@V_2_O_5_//AC supercapacitor from 0 to 1.8 V, c) cycling behavior at the current rate of 10 C, d) Ragone plots of the asymmetric V_2_O_5_//AC and PPy@V_2_O_5_//AC supercapacitors using 0.5 mol L^−1^ K_2_SO_4_ solution as the electrolyte, adapted with permission.^[^ ^55^ ^]^ Copyright 2012, Wiley‐VCH, e) TEM images of CTAB−Sn(IV)@Ti_3_C_2_; Inset in (e): lateral size distribution of the anchored Sn(IV) nanocomplex, f) Charging process of CTAB−Sn(IV)@Ti_3_C_2_//AC LIC, g) typical charge−discharge curves of CTAB−Sn(IV)@Ti_3_C_2_//AC LIC, and h) long‐term cycling performance of LIC at 2 A g^−1^; Inset in panel (h): charge−discharge curves at 2 A g^−1^, adapted with permission.^[^ ^56^ ^]^ Copyright 2017, American Chemical Society, i) Corresponding HRTEM image of crystallized V_2_O_5_ nanosheets with SEAD pattern, j) galvanostatic charge–discharge voltage profile, k) cycling stability of the flexible V_2_O_5_ –ECF//ECF ASC device, and l) photograph of LED powered by the V_2_O_5_–ECF//ECF ASC device, adapted with permission.^[^ ^57^ ^]^ Copyright 2015, Wiley‐VCH.

Accordingly, Qunting Qu et al. worked on this type of system. Their report indicates that the TEM images (**Figure** **4**a) confirm the core‐shell structure of the PPy@V_2_O_5_ nanocomposite, with a uniform PPy film on V_2_O_5_ nanoribbons. Flanges in the PPy shell correlate with leaf‐like nanoparticles, as seen in SEM images. For the supercapacitor assembly, activated carbon serves as the cathode, with 0.5 mol L^−1^ K_2_SO_4_ used as an electrolyte and cycling from 0 to 1.8 V (Figure 4b). The PPy@V_2_O_5_//AC supercapacitor exhibits remarkable cycling stability, with ≈5% capacitance loss after 10000 cycles (Figure 4c). The PPy shell prevents vanadium dissolution, evident in the colorless electrolyte. Additionally, the volume of the PPy coating buffers changes in V_2_O_5_, enhancing structural stability. Ragone plots (Figure 4d) reveal superior performance for PPy@V_2_O_5_//AC, with an energy density of 42 Wh kg^−1^ and impressive rate behavior at 2.5 kW kg^−1^, competing favorably with various batteries and marking PPy@V_2_O_5_//AC as a promising high‐performance energy storage candidate.^[^ ^55^ ^]^

Similarly, Jianmin Luo et al. reported that the TEM image of CTAB−Sn(IV)@Ti_3_C_2_ (Figure 4e) shows dot‐shaped Sn(IV) nanocomplexes, measuring 2 to 5 nm in diameter, uniformly distributed in the CTAB@Ti_3_C_2_ matrix. STEM imaging confirms the well‐defined layered structure and elemental mapping verifies Sn(IV) intercalation. Utilizing its outstanding energy storage capabilities, CTAB−Sn(IV)@Ti_3_C_2_ serves as the anode for lithium‐ion capacitors (LICs), accompanied by a commercial activated carbon electrode (Figure 4f). ASCs demonstrates a capacity of 34 mAh g^−1^ at 0.2 A g^−1^. Charge/discharge curves (Figure 4g) show nearly linear behavior, with specific capacitance values of 51, 42, 34, 33, and 25 F g^−1^ at 0.2, 0.5, 1, 2, and 5 A g^−1^, respectively, based on the weight of CTAB−Sn(IV)@Ti_3_C_2_ and activated carbon. The CTAB−Sn(IV)@Ti_3_C_2_//AC LIC exhibits consistent cycling performance, retaining 71.1% of its capacity after 4000 cycles at 2 A g^−1^ (Figure 4h), with nearly 100% Coulombic efficiency, affirming its stability as a high‐performance lithium‐ion capacitor anode.^[^ ^56^ ^]^

Another example based on this system was explored by Li et al. The HRTEM image of V_2_O_5_ nanosheets (Figure 4i) reveals lattice fringes corresponding to the (110) planes, emphasizing their hierarchical structure formation linked to VOT concentration. The galvanostatic charge–discharge profile (Figure 4j) verifies the pseudocapacitance contribution, which is consistent with CV results. Figure 4k depicts the V_2_O_5_–ECF//ECF device stability over 10000 cycles, with about a 10.7% decay in specific capacitance. Notably, the ASEC device powers red LEDs in 35 s (Figure 4l), showcasing the potential of V_2_O_5_–ECF//ECF as a high‐performance energy storage solution with its innovative, flexible integrated electrode architecture.^[^ ^57^ ^]^ Thus, recently reported hybrid capacitors with cell configuration and electrochemical performance are reviewed in Table S3 (Supporting Information).^[^ ^58^, ^59^, ^60^, ^61^, ^62^ ^]^

#### 3.3.1 Metal‐Ion Capacitor

Premetalation is essential for high‐performance charge storage devices. In metal‐ion capacitors, the cathode facilitates anion intercalation with carbon‐based materials, like the activated carbon anode. Electrolyte ions are reservoirs for cations and anions stored in their respective electrodes. Charge storage at the positive electrode involves Faradaic reactions with anions (Equation (6)), while the negative electrode stores charge through electrochemical double‐layer formation with cations (Equation (7)).

(6) $$\mathrm{Positive}\;\mathrm{electrode}:\;C+xA^{-}⇌\mathrm{AxC}+xe^{-}$$

(7) $$\mathrm{Negative}\;\mathrm{electrode}:\;\mathrm{AC}+xC^{+}+xe^{-}⇌\mathrm{AC}//\mathrm{xC}$$

This hybrid configuration exhibits a key distinction: the average concentration of charge carriers changes during the charge–discharge cycle due to a both‐in/both‐out mechanism. Ideal operation entails cation intercalation at lower potentials and anion insertion at higher potentials. In a liquid electrolyte, the potential window is determined by its lowest unoccupied molecular orbital (LUMO) and highest occupied molecular orbital (HOMO). A stable cell is achieved when the electrochemical potentials (µ) of both the negative and positive electrodes align with the LUMO and HOMO of the electrolytes, respectively. Ensuring the negative electrode Fermi level is below the LUMO and the positive electrode Fermi level is above the HOMO prevents electrolyte reduction and oxidation. Selecting a stable electrolyte for high potential is also crucial, especially due to anion intercalation at elevated potentials.^[^ ^63^, ^64^ ^]^

**Figure 5:**
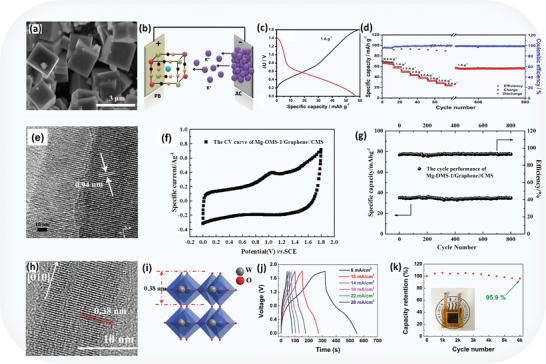
Capacitive activity in Faradaic ASCs: a) scanning electron microscopy (SEM) image, b) KIC fabricated using a Prussian blue positive electrode and an activated carbon negative electrode in K_2_SO_4_ aqueous electrolyte, c) galvanostatic charge/discharge curves ranging from 0 to 1.6 V, d) various specific capacities at different current densities and cycling behavior at a current density of 1 A g^−1^, adapted with permission.^[^ ^65^ ^]^ Copyright 2017, Elsevier. e) HRTEM image of Mg‐OMS‐1/graphene powder material, f) CV curve and g) cycle performance of Mg‐OMS‐1/graphene//CMS battery capacitor adapted with permission.^[^ ^66^ ^]^ Copyright 2019, American Chemical Society, h) HRTEM image of a single W_18_O_49_NW, i) atomic structural model of monoclinic W_18_O_49_ showing WO_6_ octahedra, j) galvanostatic charge/discharge curves of the ASCs, and k) long‐term cycling stability of the ASCs charged and discharged at 14 mA cm^−2^adapted with permission.^[^ ^67^ ^]^ Copyright 2017, Wiley‐VCH.

In the investigation by Lei Zhou et al., SEM imaging (**Figure** **5**a) revealed a cubic morphology of the prepared PB sample, averaging 2–3 µm in size. The KIC demonstrated impressive electrochemical performance using a Prussian blue positive electrode and activated carbon negative electrode in a K_2_SO_4_ electrolyte (Figure 5b). Galvanostatic charge/discharge tests (Figure 5c) showed a specific discharge capacity of 66 mAh g^−1^ at 0.5 A g^−1^, maintaining remarkable reversible capacities at various current densities, along with excellent cycling stability (Figure 5d). This underscores the KIC's robustness and potential for practical applications.^[^ ^65^ ^]^

Similarly, Hongyu Zhang et al. observed distinct lattice fringes in the HRTEM image (Figure 5e), confirming crystallinity with an interlayer distance of 0.94 nm corresponding to the (100) plane. Electrochemical tests of the Mg‐OMS‐1/graphene//CMS device demonstrated both battery and capacitor characteristics, as highlighted by the anodic peak at 1.1 V and the half‐rectangular shape of the CV curve (Figure 5f). The device's cycling stability, depicted in Figure 5g at a current density of 100 mA g^−1^, with 98.6% capacity retention after 800 cycles, indicates its potential practical applicability.^[^ ^66^ ^]^

As reported by Kerui Li et al., the intense (010) and (020) peaks indicate crystal growth along the [010] direction, corroborated by the HRTEM image (Figure 5h). Clear lattice fringes corresponding to the (010) planes and ordered layered nanowires with wide lattice spacing (Figure 5i) are ideal for Al ion intercalation/deintercalation. Specific capacitance was assessed through galvanostatic charge/discharge tests at different current densities (Figure 5j). The ASCs exhibited remarkable cycling stability, retaining 95.9% of their initial capacitance after 6000 cycles (Figure 5k). The consistent structural characteristics and electrochemical performance of W_18_O_49_ underscore its potential for advanced energy storage applications.^[^ ^67^ ^]^ In addition, lately reported metal‐ion capacitors with cell configuration and electrochemical performance are collected in Table S3 (Supporting Information).^[^ ^68^, ^69^, ^70^, ^71^, ^72^ ^]^

#### 3.3.2 Redox‐Electrolyte Capacitor

For enhancing ion adsorption, one strategy involves increasing the surface area of carbon materials, though this often reduces electrode tap density. Altering carbon with pseudocapacitive materials merges capacitance but at the cost of conductivity and stability. Introducing soluble redox mediators into inert electrolytes can boost the energy density in EDLCs. However, current redox mediators typically affect only one electrode, thereby limiting voltage and energy density. Utilizing dual redox mediators for each electrode separately addresses this issue but introduces additional complexity. A more desirable solution is to find an ambipolar redox mediator that simultaneously contributes capacitance to both electrodes.^[^ ^73^, ^74^ ^]^

**Figure 6:**
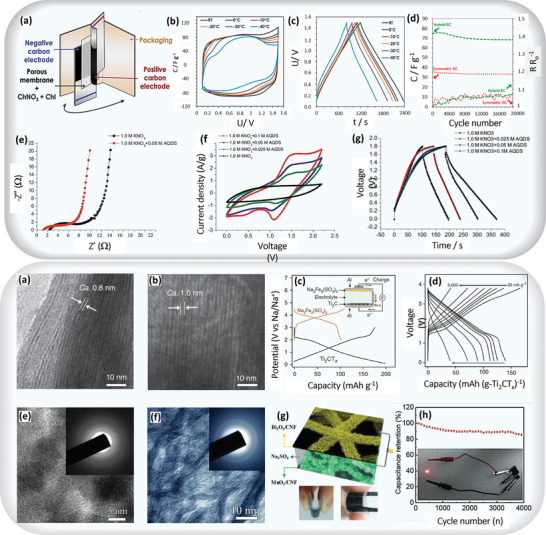
(Top panel) Capacitive activity in redox‐electrolyte ASCs: a) hybrid pouch cell components, b) CVs at 2 mV s^−1^, c) GCPL curves (‐)KAC/PAC(+) hybrid cell using 5 mol kg^−1^ ChNO_3_ + 0.5 mol kg^−1^ ChI from room temperature (RT) to −40 °C, d) Specific cell capacitance and relative resistance recorded at RT during 20000 galvanostatic (0.5 A g^−1^) cycles from 0.1 to 1.5 V for symmetric (‐)KAC/KAC (+) capacitor in 5 mol kg^−1^ ChNO_3_ (red dotted lines) and asymmetric (‐)KAC/PAC (+) hybrid capacitor in 5 mol kg^−1^ ChNO_3_ + 0.5 mol kg^−1^ ChI (green dashed lines) adapted with permission.^[^ ^75^ ^]^ Copyright 2019, Elsevier. e) Nyquist plots of ASC electrode in 1.0 m KNO_3_ or 1.0 m KNO_3_ with 0.05 m AQDS, f) cyclic voltammograms of ASC symmetric supercapacitor in electrolytes of 1.0 m KNO_3_ or 1.0 m KNO_3_ with different concentrations of AQDS at a scan rate of 20 mV s^−1^, and g) typical charge‐discharge curves of ASCs symmetric supercapacitor in electrolytes of 1.0 m KNO_3_ or 1.0 m KNO_3_ with various AQDS concentrations adapted with permission.^[^ ^76^ ^]^ Copyright 2015 Elsevier.^[^ ^75^, ^76^ ^]^ (Bottom panel) a) Transmission electron microscopy (TEM) image of the pristine Ti_2_CT_x_, b) TEM image of activated Ti_2_CT_x_ after the first CV, c) charge/discharge curves of Ti_2_CT_x_ and alluaudite Na_2_Fe_2_(SO_4_)_3_ versus Na/Na^+^; the specific currents are 30 and 6 mA g^−1^, respectively, d) charge/discharge profiles at various rates adapted with permission.^[^ ^86^ ^]^ Copyright 2015, Springer Nature, e) HRTEM image (inset: SAED pattern) of the flower‐like Bi_2_O_3_, f) HRTEM image (inset: SAED pattern) of the flower‐like MnO_2,_ g) schematic illustration of the structure of the assembled flexible ASC, and h) cycling performance of the ASC device at 6 mA cm^−2^ over 4000 cycles dapted with permission.^[^ ^87^ ^]^ Copyright 2014, Wiley‐VCH Verlag GmbH & KGaA, Weinheim.

For instance, in the study by Ying Tian et al., the current collector material was vacuum‐dried at 120 °C for 12 h and subsequently cooled. Two carbon electrodes, each measuring 5 cm × 6 cm, were immersed in the electrolyte, surrounded by a 7 cm × 7 cm glassy microfiber separator. The assembly, containing 1.0 ml of electrolyte, was sealed in an aluminum pouch and degassed for 5 s using a vacuum packer (**Figure** **6**a, Top panel). An asymmetric hybrid cell in ChNO_3_ + ChI exhibited a moderate decline in capacitive current and discharge time at −30 and −40 °C (Figure 6b,c, Top panel). Both capacitors, subjected to prolonged cycling at 0.5 A g^−1^ up to 1.5 V, demonstrated good performance. The hybrid capacitor maintained a high capacitance (75 F g^−1^) for up to 3000 cycles, decreasing slightly to 92% after 20000 cycles (Figure 6d, Top panel). The symmetric capacitor gradually declined to 92% of its initial capacitance after the same number of cycles, underscoring its long‐term durability.^[^ ^75^ ^]^

Similarly, Patryk Przygocki et al. proposed that Nyquist plots, as shown in Figure 6e (Top panel), reveal the electrochemical behaviors of the activated carbon electrode in AQDS‐KNO_3_ and KNO_3_ electrolytes, demonstrating ideal capacitor Warburg impedance. KNO_3_‐AQDS exhibits lower inner and charge transfer resistance, suggesting enhanced electrode/electrolyte interaction and improved properties of the activated carbon electrode. Figure 6f (Top panel) displays CVs of symmetric two‐electrode cells in KNO_3_ and KNO_3_‐AQDS, showing rectangular shapes typical of electrical double‐layer capacitors and redox humps indicative of AQ/H_2_AQ reactions, respectively. The redox additive significantly enhances the current response, with increased AQDS concentration leading to higher specific capacitance and improved energy storage. Figure 6g (Top panel) illustrates galvanostatic charge/discharge curves for ASCs with KNO_3_ and KNO_3_‐AQDS within the 0.0 – 1.8 V range. KNO_3_‐AQDS shows pseudocapacitive behavior, while KNO_3_ displays an ideal double‐layer capacitor response. Higher concentrations of AQDS result in longer charge‐discharge periods compared to increased CV capacitance values.^[^ ^76^ ^]^ In addition, currently reported redox‐electrolyte capacitors with cell configuration and electrochemical performance are reported in Table S3 (Supporting Information).^[^ ^77^, ^78^, ^79^, ^80^, ^81^ ^]^

#### 3.3.3 Battery Type ASCs

The rise of hybrid electric vehicles (HEVs) and electric vehicles (EVs) underscores the demand for energy storage devices that blend the high‐power density of supercapacitors with the high energy density of lithium‐ion batteries. Hybrid battery capacitors integrate these materials in a single unit cell.^[^ ^82^, ^83^ ^]^ They have attracted attention due to their potential to achieve high energy and power density. Aqueous ASCs are promising for electronic devices, offering benefits in terms of energy density, power density, specific capacitance, internal resistance, and cycle durability. Being cost‐effective and eco‐friendly, ASCs provide a safer alternative to conventional supercapacitors with organic electrolytes, surpassing their energy density due to lower specific capacitance. Hybrid battery capacitors originated in 2001, with an asymmetric design featuring a Li_4_Ti_5_O_12_ anode and activated carbon cathode.^[^ ^84^ ^]^ Hybrid battery capacitors employing a Li_4_Ti_5_O_12_ anode are an effective solution to meet the dual challenge of achieving high energy and power density requirements.^[^ ^84^, ^85^ ^]^

For instance, in the study by Xianfen Wang et al., the shift in the (002) diffraction peak indicated a 2.5 Å interlayer distance expansion, as confirmed by microscopy images (Figure 6a,b, Bottom panel). This expansion is consistent with the size of bare Na⁺, suggesting Na⁺ intercalation at the electrode/electrolyte interface after desolation, exhibiting typical capacitor behavior. Reversible sharp cathodic/anodic peaks at ≈2.3 V in each cycle signify Na⁺ ion intercalation. In Na‐ion hybrid capacitors, Ti_2_CT_x_, with a 4:1 mass balance, shows promising high‐power performance in a prototype full cell. This cell achieves high energy and power densities using Ti_2_CT_x_ as the negative electrode and alluaudite Na_2_Fe_2_(SO_4_)_3_ as the positive electrode. Charge–discharge profiles (Figure 6c, Bottom panel) display reversible capacities of 90 and 40 mAh g⁻¹ at high rates of 1000 and 5,000 mA g⁻¹ (based on Ti_2_CT_x_ weight). The pseudocapacitive negative electrode enables the overcoming of trade‐off limits, potentially leading to better high‐power rechargeable batteries. Despite a limited capacity (26.6 mAh g⁻¹ at a low rate) in the prototype cell due to the total electrode material weight, improving the initial Coulombic efficiency could significantly enhance the full cell's capacity (Figure 6d, Bottom panel).^[^ ^86^ ^]^

**Figure 7:**
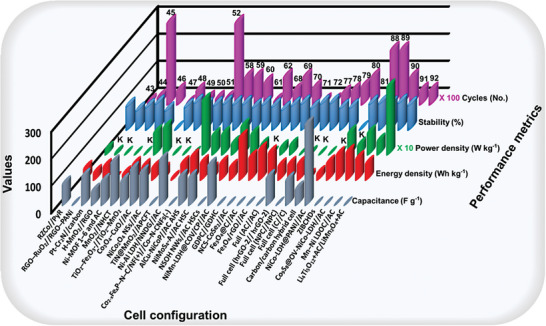
Recently reported ASCs with cell configuration and electrochemical performance.^[^ ^43^, ^44^, ^45^, ^46^, ^47^, ^48^, ^49^, ^50^, ^51^, ^52^, ^58^, ^59^, ^60^, ^61^, ^62^, ^68^, ^69^, ^70^, ^71^, ^72^, ^77^, ^78^, ^79^, ^80^, ^81^, ^88^, ^89^, ^90^, ^91^, ^92^ ^]^ 1) In graph reference numbers are put on the bars, 2) In reference number 46 energy and power density are represented in the mWhcm^−3^ and Wcm^−3^, respectively, 3) In reference number 52 capacitance, energy, and power density are represented in the F cm^−2^, mWhcm^−3^, and Wcm^−3^, respectively, and 4) In the graph “K” = 1000 is used as a factor for the concern value and more details about cell configuration (reference numbers: 72–80) is available in Table S3 (Supporting Information).

Similarly, Henghui Xu et al. reported that Bi_2_O_3_ nanoflowers, observed via TEM images, consist of interconnected sheet‐like subunits with hierarchically porous wormhole‐like nanopores (Figure 6e, Bottom panel). Limited crystallization is indicated by the dim fringe spacing in the SAED pattern. STEM and X‐ray mappings confirm the homogeneous deposition of Bi_2_O_3_ nanosheets throughout the CNF paper. MnO_2_ flowers with interconnected sheet‐like subunits and an open structure, featuring nanopores and nanocrystals, are also observed (Figure 6f, Bottom panel). The presence of nanopores enhances ion transfer, and the poor crystallinity corresponds with XRD results, as indicated by the dim fringe spacing and the SAED pattern. To evaluate the feasibility of a composite electrode, an ASC device was assembled using Bi_2_O_3_/CNF paper as the negative electrode and MnO_2_/CNF paper as the positive electrode in a 1 m Na_2_SO_4_ electrolyte (Figure 6g, Bottom panel). A 60 min deposition time for MnO_2_ was chosen to balance the areal capacitance with Bi_2_O_3_/CNF paper. The ASC device demonstrates remarkable rate capability, retaining 80% of its capacitance as the current density increases from 1.5 to 15 mA cm^−2^. It maintains electrochemical stability, with up to 85% capacitance retention even after 4000 cycles (Figure 6h, Bottom panel). The inset in Figure 6h (Bottom panel) shows a red LED lit by two ASCs in series, highlighting promising practical applications in energy storage.^[^ ^87^ ^]^ Additionally, recently reported battery/capacitors with cell configuration and electrochemical performance are summarized in **Figure** **7**, and Table S3 (Supporting Information).^[^ ^88^, ^89^, ^90^, ^91^, ^92^ ^]^

## 4 Discussion of Electrode Materials Used in ASCs

### 4.1 Negative Electrode Material for ASCs

#### 4.1.1 Materials Based on Carbon

**Figure 8:**
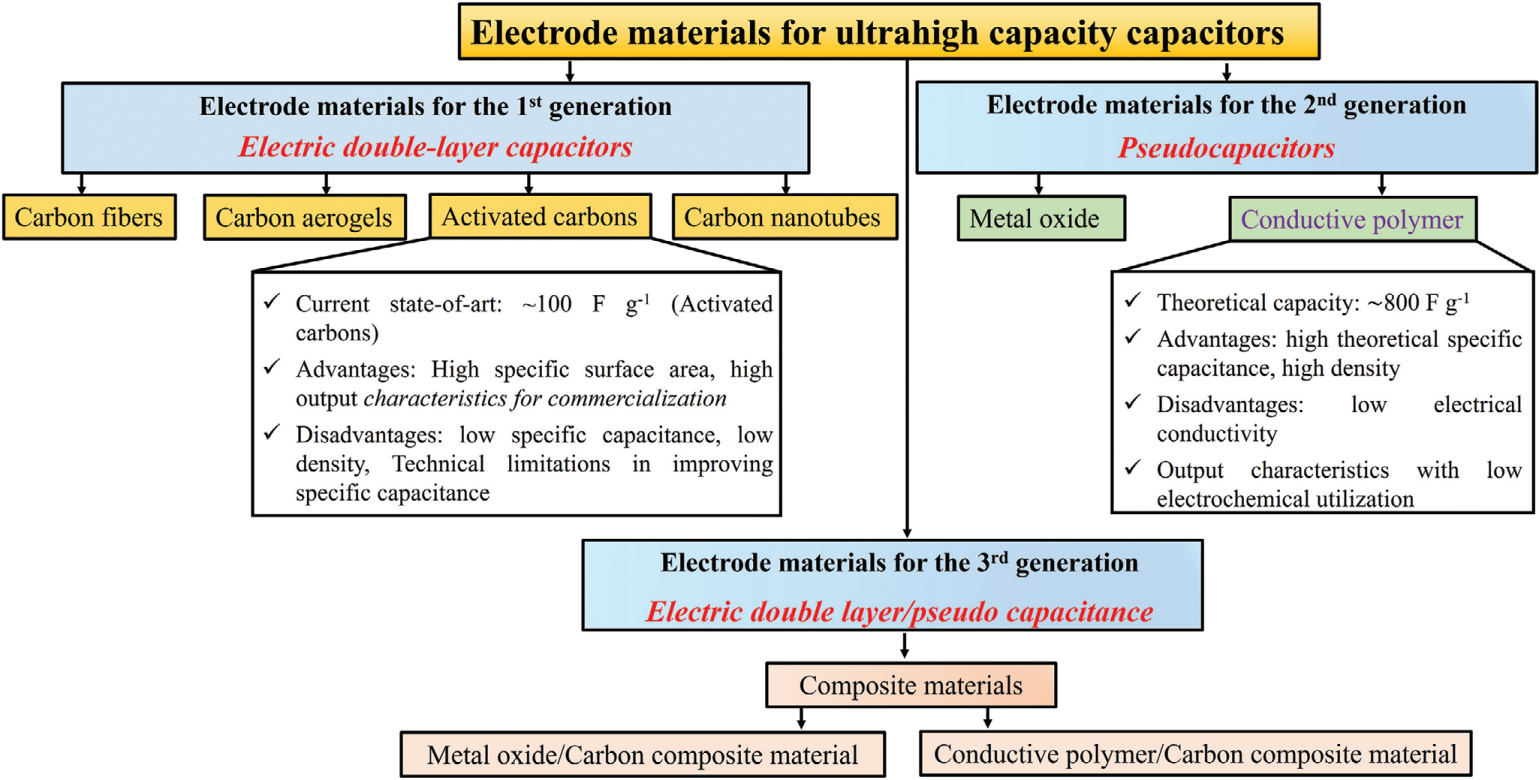
Electrode materials for first, second, and third‐generation supercapacitors.^[^ ^100^, ^101^, ^102^ ^]^

The various carbon materials, including activated carbon, CNTs, and Graphene Oxide (GO), are utilized as electrode materials in EDLCs. The capacitance of EDLCs is directly linked to the surface area of the active material. Among these, carbon aerogels, noted for their remarkable surface area and low electrochemical resistance, garner attention due to their chemical linkage with the current collector, eliminating the need for additional binders. CNTs, employed as supercapacitor (SC) electrodes, exhibit commendable mechanical strength, tubular network, low mass, and low resistivity but face limitations in practical EDLC applications due to their relatively low surface area and high synthesis cost (<500 m^2^ g^−1^).^[^ ^93^, ^94^, ^95^ ^]^ In addition, the summary of the electrode material is represented in **Figure** **8**.

##### Activated Carbons (AC)

AC is an adaptable carbon form with lightweight, and high electrical conductivity, making it a suitable material for various applications in chemical, petrochemical, food industries, and automotive as well as electrode materials in energy storage. AC effectively stores energy with high power ability and longer life span via enhancing surface wettability due to nanostructured form and doping of heteroatoms. Current research exploring AC from various sources like biowaste‐derived, human hair, etc. It shows diverse morphologies and textures. Moreover, low cost, simple bulk production, environmentally friendly, and non‐toxicity, further increase its efficacy. The characteristics of AC are affected by various components like heating rate, activating agent, activation temperature, etc. In addition, heteroatom functionalities improve ion wettability, and oxygen functionalities accelerate the physisorption of ions which is beneficial as electrode material in ASCs.^[^ ^96^, ^97^ ^]^

##### Carbon Nanotubes (CNTs)

CNTs (single wall or multiwall) are vastly employed as electrodes in supercapacitors because of their properties like mechanical flexibility, higher electrical conductivity, and thermal characteristics. CNTs possess both pseudo‐capacitance and EDLC‐type behavior due to their tubular structure which offers strong backing for electrochemical active materials through local surface area. Still, the CNTs have moderate energy density and capacitance as compared to other carbon‐based material supercapacitors. Therefore, to enhance the electrochemical performance of CNTs, they can combine with different transition metal oxides (MOs). Thus, more research focus is required to investigate through advanced construction and post‐treatment systems for completely and efficiently applicable these materials in supercapacitors as electrodes.^[^ ^98^, ^99^ ^]^

##### Graphene Oxide (GO)

Graphene, characterized by a honeycomb‐like crystal lattice formed by a 2D planar sheet of densely packed sp^2^ carbon‐bonded atoms, draws attention to its electrical and mechanical properties. Also, it has a π‐electron cloud below and above the side of the plane with ≈0.142 nm carbon‐carbon bond length. Interestingly, ≈1 mm thick graphite crystal consists of ≈3 million stack layers of graphene and it possesses different forms such as fullerene (0D), CNT (1D), and graphite (3D). In addition, graphene has high electrical mobility of charge carrier, high mechanical strength, low weight, and high thermal conductivity of ≈250000 cm^2^ V⁻¹ s⁻¹, ≈1 TPa, 0.77 mg m⁻^2^ and ≈5000 W m⁻¹ K⁻¹, respectively. Moreover, highly conductive graphene sheets can be obtained by reducing GO chemically. The GO comprises carbonyl groups, epoxy, and hydroxyl groups which can be reduced by deoxygenation. Thus, this reduction offers wrinkled and defect‐rich graphene which is appropriate for electrochemical applications. Furthermore, graphene has been vastly applied in various applications such as nanomechanical devices, nanoelectronics, conductive nanocomposite fillers, optoelectronics, electrochemical sensing, conversion materials, and energy storage.^[^ ^103^, ^104^ ^]^

However, graphite derivatives, like graphene, display remarkable characteristics, including a high Young's modulus, carrier mobility, large spring constant, and surface area. Recent interest also extends to GO nanosheets, synthesized through chemical exfoliation. This allows for the development of hybrid electrode materials with tailorable electronic properties and high stability, facilitated by various functional groups attached to GO.^[^ ^105^, ^106^, ^107^ ^]^ Moreover, the analysis of carbon electrode material is shown in **Table** **1**.^[^ ^108^, ^109^, ^110^, ^111^, ^112^, ^113^, ^114^, ^115^, ^116^, ^117^, ^118^, ^119^, ^120^ ^]^

**Table 1:** Analysis of Carbon Electrode Materials.

Raw material	Activation process	Unit cost/Surface area	Note
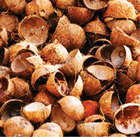 Coconut shell (< 0.75$ kg^−1^)	Steam activation (Low Cost)	≈13 – 15 $ kg^−1^/ ≈1800 m^2^ g^−1^	Batch control required Low quality (≈70–80 F g^−1^)
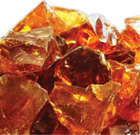 Phenolic resin (> 6 $ kg^−1^)	Steam activation (Low Cost) Alkaline activation (High Cost)	≈30 – 38 $ kg^−1^ / ≈1300 – 1800 m^2^ g^−1^ ≈52 – 75 $ kg^−1^/ ≈1800 – 2200 m^2^ g^−1^	Medium price, difficult to control, Low quality (≈70–90 F g^−1^)
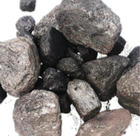 Coal (< 1.5 $ kg^−1^)	Alkaline activation (High Cost)	≈37 – 60 $ kg^−1^/ ≈2000 m^2^ g^−1^	High price, high purity, high quality (≈90–110 F g^−1^)
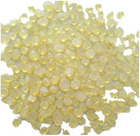 Precursor candidate material (non‐phenolic polymer) (<1.13 $ kg^−1^)	Steam activation (Low Cost) Primary hydrocarbons yield 45% Secondary activation yields 30%	≈19 – 22 $ kg^−1^/ ≈1700 – 2300 m^2^ g^−1^	High price, high purity, high quality (≈90–120 F g^−1^) High nitrogen content

### 4.2 Positive Electrode Material for ASCs

#### 4.2.1 Pseudocapacitive Materials

As defined by Conway, pseudocapacitance originates from fast and reversible redox reactions at electrode surfaces, distinguishing it as a Faradaic process in contrast to EDLC. The capacitance is attributed to the unique relationship between charge acceptance (ΔQ) and potential change (ΔV), expressed as dQ/dV, effectively reflecting the capacitance value. In the case of pseudocapacitive materials, this pseudocapacitance typically occurs on or near the electrode surface.^[^ ^121^ ^]^

Despite exhibiting redox behavior akin to a battery, pseudocapacitance is characterized by nearly linear GCD curves and quasi‐rectangular CV curves, indicative of rapid Faradaic reactions. The distinction between non‐Faradaic and Faradaic processes demarcates pseudocapacitance from traditional capacitance, justifying the prefix “pseudo” in pseudocapacitance. Materials such as metal oxides, including ruthenium oxide, manganese oxide, and vanadium nitride, along with conducting polymers like polyaniline, other transition metal oxides, carbon‐based heteroatoms, and nanoporous carbons, typically demonstrate pseudocapacitance.^[^ ^122^, ^123^ ^]^

##### Metal Oxide (MO)

Since, carbon‐based materials show chemical stability, higher surface area, and good conductivity but they have lower capacitance due to their limited accessibility of ions. Thus, the MOs are reliable candidates used as positive electrode material in supercapacitors due to their redox behavior and unique electronic configuration. However, as compared to carbon‐based materials MOs show up to 10–100 times higher capacitance due to their fast redox reaction behavior at the interface of electrolyte and electrode. For example, IrO_2_ and RuO_2_ have good conductivity, power density, and higher specific capacitance but they are more toxic and very costly. Therefore CO_3_O_4_, NiO, and MnO_2_ MOs are preferred as a good alternative candidate for the supercapacitors due to their environmentally friendly, easy availability, redox behavior, and low cost. Nevertheless, MOs have disadvantages such as poor conductivity, low surface area, and stability issues because of volume variations during electrochemical study. In addition, MO properties can be controlled over diverse synthesis processes such as electrochemical deposition, sol‐gel, hydrothermal, and pyrolysis which are influenced by their shape, size, and morphology. Thus, specifically tuning of MO morphology can highly influence the electrochemical performance.^[^ ^124^, ^125^ ^]^

##### Conductive Polymers (CP)

Conducting polymers (CPs) are designed by the electrochemical or chemical oxidation of monomers. Which introduces counterions like chloride or dopants into the polymer spine. CPs like polyaniline, polypyrrole, and polythiophene are employed in supercapacitors as electrode materials. Nevertheless, they possess low cycle life as compared to carbon‐based supercapacitors because of structural variations due to doping or de‐doping. While increasing dopant levels can boost specific energy, it also raises costs and causes volume changes, weakening electrode strength over time. However, to increase the life span of CP‐based electrodes it is necessary to combine the ionic liquid electrolyte with CPs, studies show.^[^ ^126^, ^127^, ^128^ ^]^

### 4.3 Composite Materials for ASCs

The supercapacitor is a futuristic charge storage device that allows high energy and power density. Second‐generation or hybrid supercapacitors improved and enhanced electrochemical performance by comprising metal oxides/hydroxides, conducting polymers, and carbon‐based hybrid materials. AC provides good electrical conductivity and has a high surface area. Meanwhile, MOs have a redox reaction mechanism that offers high capacitance but moderate durability. However, preparing composite/hybrid materials can overcome the drawbacks of individuals and help to enhance conductivity, life span, and capacity.^[^ ^129^, ^130^, ^131^ ^]^

#### 4.3.1 Metal Oxide/Carbon Composite Material (MO/CC)

The composite electrodes offer high electrochemical performance. For instance, CNTs united with MOs like NiO, MnO_2_, RuO_2_, Co_3_O_4_, etc. These composite materials enhance electrochemical performance due to the sturdy interface of MOs with CNTs, easy ion diffusion in mesoporous which are formed due to a 3D network, and high flexibility. These properties enable enhanced cycling performance and the 3D network decreases resistance and helps to enhance power density. For example, a composite of RuO_2_ MWCNTs increases the hydrophilicity of MWCNTs and ultimately helps to improve charge storage. Therefore, joining MOs with carbon‐based materials allows electrical conductivity, chemical stability, mechanical strength, and large surface area. Ultimately these hybrid electrodes carry long cycle life with high specific energy and power due to synergistic effects in between carbon‐base materials and MOs.^[^ ^132^, ^133^ ^]^

#### 4.3.2 Conductive Polymer/Carbon Composite Material (CP/CC)

An electrode constructed from CPs possesses high theoretical capacitances but still has low energy density. Thus, there is a need to combine CPs with MOs to produce composite electrode materials to enhance electrochemical performance. However, establishing MOs‐CPs composites expands redox site accessibility and electrical conductivity leading to high‐performance supercapacitors. Generally, the electrochemical deposition technique is used to deposit MOs onto the CP electrode through a half‐cell system. This system regulates electrochemical parameters and controls the size and structural morphology of the particle. MOs‐CP composites deliver high electrical conductivity, short ion‐electron transportation routes, and high surface area leading to enhanced electrochemical performance. Moreover, ternary composites prepared with CP, MO, and carbon can help to further improve the specific capacity and durability of energy storage devices. For example, the polyaniline (PANI), MnO_2_, and graphene composite display the fine results. The PANI@MnO_2_/graphene ternary composite shows a specific capacitance of 875.2 F g^−1^, which is improved than that of graphene, PANI, and PANI/graphene at the 0.2 A g^−1^.^[^ ^134^ ^]^

In addition, carbon‐based material has high and comparable electrical conductivity. Besides, defects and impurities such as amorphous carbon and catalysts in metallic nanotubes reduced the transportation of electrons. Thus, using CNTs (single, double, or few‐walled) with fiber and anneal at 2000 °C expressively improves conductivity. Moreover, tunning the defects and fiber structures by altering pores, voids, and tube dislocation can help to hinder contact resistance. For instance, Zhao et al. obtained a high electrical conductivity of 6.7 × 10⁴ S cm^−1^ with I‐doped CNT fibers. Due to the doping of iodine polyiodide chains are formed and significantly increase the concentration of holes.^[^ ^134^, ^135^, ^136^, ^137^ ^]^ Thus, the preparation of composite material like carbon base material with MOs and CPs is better for state‐of‐art electronic devices because of high conductivity, and their flexibility. These composites suggest decent capacitance with improved high energy and power densities. Also, offers an enlarged surface area and upgraded charge storage ability.^[^ ^133^, ^138^, ^139^, ^140^ ^]^

## 5 Performance Metrics of ASCs

Developing standardized metrics for various evaluations of electrochemical performance materials is crucial. Specifically, performance metrics for nickel and cobalt‐based electrode materials are often reported exaggeratedly. Therefore, these materials report their performance as specific capacity in C g^−1^ or mAh g^−1^ instead of capacitance in terms of thousands of Farads per gram (F g^−1^). In addition, conducting polymers such as polyaniline, polypyrrole, and poly(3,4‐ethylenedioxythiophene) are assessed as pseudocapacitive materials due to their capability to store charge via intercalation and deintercalation of electrolyte ions. These conductive polymers display specific capacitances that are comparable to or even better than those of several pseudocapacitive metal oxides and demonstrate effective performance about both positive and negative potentials. In hybrid composite electrodes, carbon materials based on heteroatoms boost electrochemical performance while the pseudocapacitance is contributed by metallic hydroxides/oxides or conductive polymers. Moreover, researchers are investigating diverse nanoporous carbon materials such as graphene, carbon nanotubes, and carbon‐based ternary hybrids, influencing the enriched performance of composite hybrids. Therefore, electrodes based on these materials are also able to report capacity in C g^−1^ or mAh g^−1^.^[^ ^141^, ^142^, ^143^, ^144^, ^145^, ^146^, ^147^, ^148^ ^]^ Furthermore, it is important to highlight the significance of reporting volumetric electrochemical parameters for solid‐state ASC devices, considering the negligible mass loading of active material compared to the overall device weight.^[^ ^149^ ^]^

### 5.1 Energy Density

Energy density is a key parameter in assessing an electrochemical supercapacitor. Specifically, a more widely adopted and practical metric, known as specific energy density, takes precedence in practical applications. This metric is defined as follows:

(8) $$E_{m\;}=\frac{1\;(C_{m\;}.\;{V^{2}}_{\mathrm{sc}\;})}{2M}$$

where E_m_ represents the specific energy density in Wh kg^−1^, C_m_ denotes the double‐layer capacitance of the supercapacitor device, M denotes its mass, and Vsc denotes the achieved maximum voltage. This is the primary metric for evaluating the energy density of double‐layer supercapacitors in practical scenarios. Moreover, the performance metrics specific energy of the ASCs can be further calculated by applying the following equation.

(9) $$E_{m}=\frac{i\;}{3.6\;(m_{+}+m_{-})}\;\;\int Vdt$$

where E_m_ is the specific energy (Wh kg^−1^), i is the applied current (mA), m_+_ is the mass of the positive electrode (mg), m_‐_ is the mass of the negative electrode (mg), and ∫Vdt is the area below the discharge curve.^[^ ^150^, ^151^ ^]^

#### 5.1.1 Factors Affecting Energy Density

Specific energy density is significantly influenced by material selection. Variations in electrolytes impact cell voltage due to differing voltage windows. Electrode materials, characterized by distinct porosities and particle sizes, contribute to varied capacitances. Therefore, it is important to choose current collector materials that are highly conductive, lightweight, and stable. The interaction between electrolyte ions and the electrode layer can also impact energy density by altering differential capacitance.^[^ ^152^ ^]^ Consequently, at a commercial scale, it may be more practical to establish volumetric energy density by considering the overall volume occupied by the device. This includes the electrodes and other essential components like current collectors, separators, connectors, and packaging.^[^ ^153^ ^]^

### 5.2 Power Density

The power (P) denotes energy expenditure over time. When evaluating capacitor power, it is crucial to consider their representation in circuits with an external load resistance (R). Internal capacitor components contribute to equivalent series resistance (ESR), influencing discharge voltage. At matched impedance (R = ESR), the maximum power (P_max_) is determined by following Equation (10).

(10) $$P_{\max}=\frac{V^{2}}{4\times \mathrm{ESR}}$$

This relationship indicates that power is directly proportional to the square of the voltage and inversely proportional to the ESR, which delineates constraints on maximum power. However, supercapacitors, comprising electrodes with larger surface areas (A) and thinner dielectrics, exhibit increased capacitance and energy. While maintaining low ESR, supercapacitors achieve comparable power densities. Yet, despite their greater capacitance, supercapacitors do not match the energy densities of batteries. Therefore, ongoing research is focused on enhancing the energy density of supercapacitors to bridge the gap between capacitors and batteries.^[^ ^154^, ^155^ ^]^ In addition, the specific power of ASCs can be further calculated by using the following equation.

(11) $$P_{\mathrm{ma}x}=3600\;\frac{E_{m\;}}{\Delta t}$$

where E_m_ is the specific energy (Wh kg^−1^), P_max_ is the specific power (W kg^−1^), and Δt is the discharge time (s).^[^ ^150^, ^151^ ^]^

### 5.3 Cycle Life

Cycle life is another crucial performance metric in supercapacitors. Supercapacitors are recognized for their extended cycle lifetimes, often advertised as ranging from 500000 to 1000000 duty cycles.^[^ ^156^ ^]^ However, real‐world conditions, such as those in regenerative braking systems, may involve rapid charge‐discharge cycles. The mentioned lifetimes are under ideal conditions, contrasting with practical commercial device usage. Pseudocapacitors, a subset of supercapacitors, utilize fast redox reactions for rapid charge‐discharge.^[^ ^157^ ^]^ Their advantage in using high surface area materials is offset by susceptibility to chemical side reactions, analogous to battery degradation. Supercapacitor degradation is primarily studied using impedance spectroscopy and mathematical modeling in various RC‐circuit configurations.^[^ ^157^, ^158^ ^]^

### 5.4 Capacitance/Capacity

The specific capacity for asymmetric systems with nonlinear constant current shapes can calculated by the equation as follows.

(12) $$Specific\;capacity\;\left(c\right)=\frac{\int \;i\;.\;dt}{m\;.\;3600}$$

The specific capacitance of devices with Faradaic mechanisms is determined by rearranging the above equation or stated as the following equation.

(13) $$Specific\;capacitance\;\left(\mathrm{Cs}\right)=\frac{Specific\;capacity\;.\;3600}{\Delta V}$$

where C, Cs, i, m, Δt, and ΔV, are attributed to specific capacity (Ah g^−1^), specific capacitance (F g^−1^), applied discharge current (A), the mass of active materials (g), discharge time (s), and potential window (V), respectively. These are reliable parameters of performance assessment for ASCs.^[^ ^159^ ^]^

### 5.5 Self‐Discharge

Self‐discharge in ASCs states the steady loss of stored charge without an exterior load, which influences performance consistency and trustworthiness. The crucial mechanisms behind this comprise Faradaic reactions, which are unintentional redox reactions within the electrolyte and electrode materials, degradation of electrode material, and decomposition of electrolyte caused due to a higher temperature or voltage instabilities. Moreover, electrochemical diffusion by charge redistribution due to ion movement, and thermal effects through fluctuated temperature. These phenomena or factors contribute to self‐discharge in ASCs. To attenuate self‐discharge, tactics include using sturdy materials and electrolytes, applying shielding coverings, and employing active thermal management. It is key to know and reduce self‐discharge to boost ASCs performance and durability, with current research focused on new materials, better electrolytes, and upgraded cell configurations. It is also crucial to circumvent metallic impurities and extreme cell voltages. Also, comprises drip currents which are relative to the mass of the electrode and state of charge.^[^ ^160^ ^]^

### 5.6 Voltage Holding

Voltage holding in ASCs is essential for energy storage which reveals their capacity to uphold charge and voltage on time without an external load. Major attributes comprise the leakage current and self‐discharging. Reduced self‐discharging rates suggest better retention. Aspects such as dielectric quality, electrolyte composition, electrode materials, and temperature disturb voltage‐holding retention. Stable materials and electrolytes enhance voltage‐holding retention, while higher temperatures cause retention loss. Thus, this test plays a crucial role in understanding and attenuating the leakage current of ASCs which is vital for commercial trustworthiness.^[^ ^161^ ^]^ For example, Sung Hun Jin et al. represent voltage holding tests of BL‐MS SSC devices for 8 h with 2 h interims up to 40 h and estimate specific capacitance. It shows a 77.7% specific capacitance retention across 40 h of voltage holding tests.^[^ ^162^ ^]^

## 6 Advantages and Applications of ASCs

Renewable solar and wind energy present clean, limitless power sources with considerable potential for development. However, global implementation poses challenges in integrating these energy sources into the power grid, chiefly due to intermittency and energy storage issues. Although ASCs are efficient in rapid charge and discharge processes, they encounter voltage limitations. These capacitors effectively address voltage fluctuations in renewable energy generation, yet they must operate within specific parameters. This section emphasizes the applications of ASCs in clean and environmentally friendly energy sources, underscoring their role in the sustainable energy landscape.

### 6.1 Solar and Renewable Energy Systems

**Figure 9:**
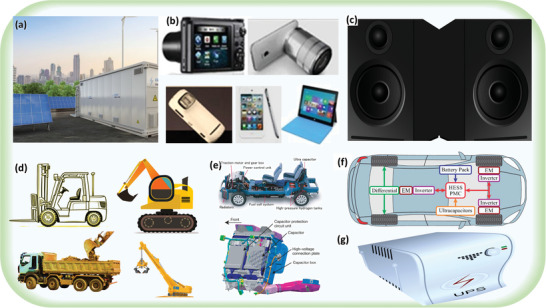
Applications of ASCs in various areas: a) renewable energy hybrid power plant, b) consumer electronic devices, c) audio system, d) industrial machinery such as forklifts, excavators, shovel trucks, and cranes, e) transportation such as hybrid EVs, adapted with permission.^[^ ^168^ ^]^ Copyright 2023, Elsevier. f) automobiles (two in‐wheel electric motors coupled to the front wheels) adapted with permission.^[^ ^169^ ^]^ Copyright 2021, Springer Nature, g) uninterruptible power supply (UPS) units.^[^ ^163^, ^164^, ^165^, ^166^, ^167^, ^168^, ^169^, ^170^, ^172^ ^]^

As a natural source, solar energy exhibits an inconsistent power supply depending on environmental conditions. Solar PV‐generated power is commonly stored in batteries, but their limited lifespan and sensitivity to fluctuations can compromise the ESS.^[^ ^163^ ^]^ In contrast, ASCs show promise in managing power demand and charging fluctuations within solar energy systems. They are being explored as alternatives to batteries to overcome these limitations. ASCs address challenges such as long cycle lifespan, rapid charge‐discharge cycles, and high‐power density, effectively handling tasks beyond the capabilities of batteries. **Figure** **9**a depicts a small‐scale test bench of a hybrid power plant, where the PV array is the primary power source, and the supercapacitor acts as the energy storage device. This setup enhances power response and improves power quality and efficiency.^[^ ^164^ ^]^

### 6.2 Consumer Electronics

Lightweight and advanced electrical energy storage devices are preferred in consumer electronic devices. Arrays of supercapacitors, alone or in combination with batteries, are used to provide energy backup for consumer electronic devices such as wireless thermometers, digital cameras, smartphones, laptops, smart toys, home theatre systems, door locks, burglar alarms, televisions, LED drivers, electronic pens, and small home appliances (Figure 9b).^[^ ^165^ ^]^

### 6.3 Audio System

Currently, ASCs are employed in various portable audio systems. As illustrated in Figure 9c, the concept aims to provide a clean and isolated power source valuable for various small audio devices and digital signal enhancements. This is achieved through dual capacitor collections, where one capacitor reserves charge while the other powers the external device.^[^ ^166^ ^]^

### 6.4 Industrial Machinery

ASCs are widely used in the industrial sector for applications such as automated guided vehicles and powering lifting operations. They benefit from regenerative braking for energy storage and offer advantages such as rapid charging, ease of operation, low costs, and long cycle life, making them economically feasible for industrial applications. Equipment like forklifts, excavators, shovel trucks, cranes, and agricultural machinery often utilize ASCs (Figure 9d). The integration of supercapacitors improves battery performance, enhancing run‐time, reliability, and overall lifespan. With their higher power density, supercapacitors enable efficient task completion in less time, reducing the reliance on numerous batteries.^[^ ^165^, ^167^ ^]^

### 6.5 Transportation

ASCs are becoming increasingly crucial in transportation, particularly as the energy source gains prominence. In EVs, the electrical conditions of the ESS are critical. Remarkably, ASCs exhibit high power density due to their lower ESR, leading to rapid acceleration in EVs (Figure 9e). However, this comes at the expense of lower energy density, affecting the overall driving range. In contrast, the internal resistance of batteries increases over time and with discharge depth, causing heat generation in the ESS. ASCs are known for their consistent ESR, minimizing heat dissipation, possessing a long life cycle, and surpassing batteries. Yet, one drawback is their high manufacturing cost. ASCs find applications in hybrid‐electric transit buses, electric braking systems for passenger cars, and hybrid vehicles. Their rapid charge‐discharge capabilities allow them to adjust energy consumption in active driving scenarios.^[^ ^168^ ^]^

### 6.6 Automotive

EVs are considered an optimal solution for future zero‐pollution mobility, characterized by zero fuel consumption, high energy efficiency, and minimal noise. To be competitive, EVs focus on low energy consumption, a satisfactory range, and acceptable driving performance. The primary obstacle lies in the ESS, which impact vehicle weight, range, energy consumption, and cost. As illustrated in Figure 9f, two in‐wheel electric motors coupled to the front wheels are integrated. The power management control of the hybrid ESS oversees power delivery and control. The analyzed hybrid ESS combines an ultracapacitor with a battery pack, optimizing performance and efficiency.^[^ ^165^, ^169^ ^]^

### 6.7 UPS Systems

ASCs play a crucial role in supplier energy storage, especially in uninterruptible power supply (UPS) units (Figure 9g). UPS systems provide instantaneous support for sensitive loads, allowing generator startups to act as emergency power devices.^[^ ^170^, ^171^ ^]^ Specific ASCs with high charge storage capacity offer maintenance‐free operation, thereby reducing environmental impacts by decreasing battery disposal needs. Unlike batteries, supercapacitors maintain performance without chemical reactions, boasting a lifespan of over 200 times longer. They can function for at least 50 years with ten charge‐discharge cycles daily. Moreover, ASCs provide near‐instantaneous charging and discharging with high specific power, and they support ≈100 times more charge‐discharge cycles than batteries. Also, ASCs require almost zero maintenance. Consequently, they significantly increase reliability and efficiency in UPS systems.^[^ ^171^, ^172^ ^]^

## 7 Conclusion and Perspectives

The progressive history of supercapacitors, beginning with the invention of the Leyden jar in the 18^th^ century, marks the early recognition of charge storage. Researchers like von Helmholtz in the 19^th^ century contributed to the modern theory of EDLC. In 1978, NEC commercialized the first non‐aqueous electrolyte‐based electrochemical capacitor, a milestone in supercapacitor development. Subsequently, in 1989, the U.S. Department of Energy initiated a study on high‐energy‐density supercapacitors, leading to collaborations with companies such as Maxwell Technologies. Since 2000, supercapacitor research has intensified due to the increased demand for high‐power, reliable, and safe energy‐storage devices.

ASCs, with their wide theoretical range, adapt to hybrid devices incorporating various electrode materials and redox‐active electrolytes. Capacitive ASCs, in contrast, feature two electrodes with capacitive properties. The energy storage mechanisms in these systems involve distinct electrode materials, redox reactions, and charge‐storage mechanisms. Various carbon materials, including CNTs, activated carbon, and graphene, are prevalent in pseudocapacitors. Despite lower conductivity, activated carbon, graphene, and other materials are widely employed in supercapacitors. Heteroatom‐containing groups enhance redox capacitance with OH^−^ ions, thus improving EDLC capacitance. A hybrid capacitor, combining pseudocapacitance and a double layer, employs polarizable (carbon) and non‐polarizable (metal or conducting polymer) electrodes. Its operation encompasses faradaic and non‐faradaic processes, resulting in electrostatic and electrochemical capacitance in distinct electrodes. The overall charge storage capacity is more by faradaic activities, where electron transfer across interfaces directs redox reactions and the faradaic current flow.

ASCs are crucial in transportation systems where the energy source is dominant. In transportation, ASCs find applications in hybrid‐electric transit. ASCs have significant applications in ESS, notably in UPS. Unlike electrochemical batteries, supercapacitors do not undergo chemical reactions, ensuring consistent performance and a lifespan nearly 200 times longer than electrochemical batteries. Their advantages in rapid charge‐discharge abilities and considerably higher charge‐discharge cycles make them preferred over traditional batteries, ensuring practically low maintenance. Thus, there is a pressing need to develop high‐energy‐density ASCs. To this end, several approaches can be beneficial for the advancement of ASCs.

1. Utilizing appropriate electrolytes like ionic liquids and non‐aqueous solutions to improve electrochemical performance, achieving compatibility between electrodes and electrolytes to enhance cell performance, and optimizing electrolyte and additive configurations for efficient reactions, especially in highly concentrated electrolytes, to ensure better potential.
2. Exploring new electrode materials with high capacitance can significantly enhance the energy storage capabilities of ASCs. Reducing costs with efficient production methods will make ASCs economically feasible and available for various energy storage applications. The composition and concentration of the electrolyte are necessary to evaluate the relationship among theoretical electric conductivity, high surface area, porosity, and electrochemically active sites. In ASC systems, bimetallic oxide/carbon composites with high conductivity and specific capacitance between the positive and negative electrodes can enhance energy density while preserving the power density constant.
3. Hybridization between supercapacitive and battery‐type electrodes may yield high power and energy density with environmental friendliness, potentially becoming a significant energy supplier for future devices. It is imperative to develop multifunctional devices with transparent, flexible, stretchable, and bendable characteristics, including sensing properties in feasible environments.
4. Various materials display multiple effects, such as energy conversion and storage properties, making them compatible with fabricating ASCs. However, this mechanism depends on case‐to‐case scenarios and remains underexplored. There is significant scope to develop novel compositions that enhance energy storage alongside energy conversion to achieve synergistic effects.
5. Safety is a critical aspect of energy storage devices and presents a considerable challenge. There is a need to fabricate asymmetric energy storage devices such as multifunctional ASCs for electrolyte leakage and thermal operational range. Commercialization and market adaptation of any system hinge on factors like performance, cost scalability, efficiency, safety, regulatory environment, and market demand.

All these studies indicate that ASC devices with favorable electrochemical activity and energy storage efficiency have been developed, but there is still room to explore these devices with additional characteristics such as flexibility and stability. Combining multiple functions into one energy storage device is an attractive prospect. Therefore, further research on optimization and smart supercapacitors will soon play vital roles in lightweight, wearable, and flexible capacitive devices.

## Conflict of Interest

The authors declare no conflict of interest.

## Supporting information

Supporting Information
