# Supplementary material for: Advancements in Asymmetric Supercapacitors: From Historical Milestones to Challenges and Future Directions
Source: Adv Sci (Weinh). 2024 Jul 9;11(34):2403172. doi: 10.1002/advs.202403172 (PMC11425848; doi:10.1002/advs.202403172)
Supplement: Supplementary file 1 — Supporting Information [file ADVS-11-2403172-s001.docx]

Supporting Information

Advancements in Asymmetric Supercapacitors: From Historical Milestones to Challenges and Future Directions

Shrikant Vaiju Sadavar^1^, Seul-Yi Lee^1^,* Soo-Jin Park^1^,*

**Principle of selecting electrodes: Electrode work function mechanism**

**
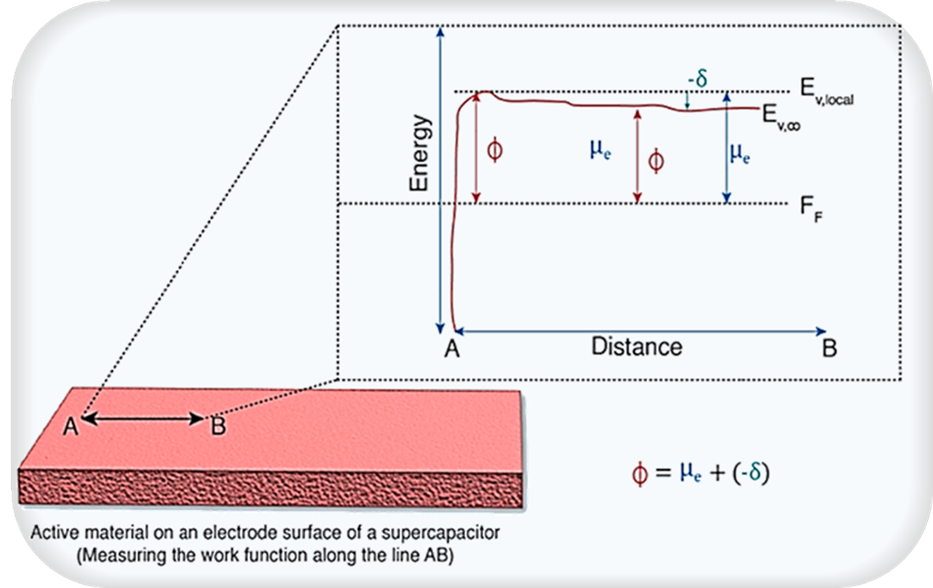
**

Figure S1. Work function mechanism; energy levels on the surface of a solid showing how the surface dipole (δ) and the electron chemical potential (μ_e_) alter the work function (Φ) of the material (E_F_ is the fermi level, E_V,local_ is the local vacuum level and E_v,∞_ is the absolute vacuum level).^[1]^

**Table S1.** Comparison of supercapacitor versus Li-ion battery based on function and characteristics.^[2-4]^

|  | **Parameters** | **Supercapacitor** | **Lithium-ion batteries** |
| --- | --- | --- | --- |
| **Function** | Charging time | 1-10 seconds | 10-60 minutes |
|  | Voltage of cell | 2.3 to 2.75 V | 3.6 to 3.7 V |
|  | Specific energy (Wh kg^-1^) | 5 | 100-200 |
|  | Specific power (W kg^-1^) | Up to 10,000 | 1000-3000 |
|  | Cost/watt | 20 $ | 0.5-1 $ |
|  | Service life | 10 to 15 years | 5 to 10 years |
|  | Charging temperature | -40ºC to 65ºC | 0 to 45ºC |
| **Characteristic** | Energy storage | Higher | Lower |
|  | Immediate power | Lower | Higher |
|  | Charge-recharge cycles | Lower | Higher |
|  | Speed of charging | Slower | Faster |
|  | Weight | Lower | Higher |
|  | Discharge curve | Long constant voltage | Linear discharge |

**Table S2.** Advantages and disadvantages of supercapacitor versus Li-ion battery, Na-ion battery, and solid-state battery.^[3, 5]^

| **Device name** | **Advantages** | **Disadvantages** |
| --- | --- | --- |
| **Supercapacitor** | High power density | Need a balancing circuit for series connections |
|  | Fast charge and discharge | Terminal voltage is directly proportional |
|  | Less possibility of short connections | Price and market delivery challenges |
|  | Stops energy flow when fully charged | Power supplies for a very short duration |
|  | Extremely small internal resistance (~ 0.01 Ω) | Highest dielectric absorption |
|  | Long cycle life |  |
|  | No emission of gas and environmentally safe |  |
| **Lithium-ion batteries** | Light weight | Risk of bursting |
|  | High energy density as compared to other batteries | As compared to other batteries high cost |
|  | Less charge loss rate | Can damage the battery while complete discharge |
|  | Maximum charge-discharge cycle rate | High temperature sensitivity (battery can damage if exposed to heat) |
|  | Due to memory loss effect no need to complete discharged | Short lifespan (maximum 2 to 3 years from the manufacturing date) |
|  | Higher operating voltage (up to 3.7 V) | Lack of availability in standard cell size (e. g. AA, C and D) |
| **Sodium-ion batteries** | Low cost | Lower energy density |
|  | Natural abundance of sodium containing precursor material (Benefit to market potential) | Poor cycle life |
|  |  | Large volume variation during sodiation and desodiation process |
| **Solid state batteries** | Can withstand low to high temperature | The mass production and manufacturing is quite complex. |
|  | Fast charging is possible | Research in progress and the perfect material for the electrolyte with an ideal ionic conductivity is yet to be found. |
|  | Longer life span |  |
|  | High degree of freedom in shape |  |
|  | Capable of delivering 2.5 times more energy density as compared to lithium-ion batteries. |  |
|  | Comparatively more durable and safer. |  |
|  | The solid electrolyte used, non-flammable, hence they are less prone to catch fire. |  |
|  | Comparatively less expensive and compact in nature. |  |
|  | The greater electrochemical stability makes them more reliable. |  |
|  | Comparatively lighter in weight. |  |
|  | The recharge rate is 4-6 times more than regular batteries. |  |
|  | Does not contain any volatile element. |  |

**Table S3.** Recently reported ASCs with cell configuration and electrochemical performance

| Cell | Electrolyte | Spe. Capacity (mAh g^−1^) | Capacitance (F g^−1^), Voltage window (V) | Energy density  (Wh kg^–1^) | Power density  (W kg^–1^) | Stabil-ity (%) | Cycle-s | Ref. |
| --- | --- | --- | --- | --- | --- | --- | --- | --- |
| 3.2 Capacitive/capacitive (Capacitive ASCs) | | | | | | | | |
| RZCo//PyR | 1 M Na_2_SO_4_ | - | 77.7, 0-1.6 | 41.8 | 188.3 | 87 | 800 | 6 |
| RGO–RuO_2_//RGO–PANi | 2 M  H_2_SO_4_ | - | −, 0–1.4 | 26.3 | 49.8 k | 70 | 2500 | 7 |
| Pt-Cr_2_N//carbon | 1 M Na_2_SO_4_ | - | 106, 1.8 | 48 | 5.170 k | 82.6 | 30000 | 8 |
| H-MnO_2_//RGO | LiCl/PVA gel | - | 50.97, 1.8 | 0.25 mWhcm^-3^ | 1.01Wcm^-3^ | 95.5 | 5000 | 9 |
| Ni-MOF 1–6 and AC | 3 M KOH |  | 87, 1.4 | 21.05 | 0.44 k | 70 | 2000 | 10 |
| MnO_2_//NHCT | Na_2_SO_4_ |  | 156.3, - | 55.8 | 803.9 | - | 5000 | 11 |
| TiO_2_–Fe_2_O_3_-  //TiO_2_–MnO_2_ | 1M Na_2_SO_4_ | - | 87.5, - | 48.6 | 1000 | 83.5 | 2000 | 12 |
| Co_3_O_4_−  CuO//AC | 6 M KOH | - | 130, 1.6 | ∼44 | 14 k | 99 | 2000 | 13 |
| NiCo_2_O_4_ NSs//AC | 6 M KOH | - | 119, 1.4 | 34.75 | 2.5 k | 86.6 | 2000 | 14 |
| TiN@MnO_2_//APCFT | LiCl–PVA | - | 0.67 F cm^−2^, 2 | 4.70 mWhcm^−3^ | 2.29 Wcm^−3^ | 89.5 | 30000 | 15 |
| 3.3 Faradaic/capacitive (Hybrid capacitor) | | | | | | | | |
| Ni-Al LDH/NNDG//AC | KOH-PVA | - | 166, 0–1.6 | 59 | 1991 | 95 | 10000 | 16 |
| Co_2-x_Fe_x_P–N–C/NF(+)//Co–N-CNFs(-) | PVA-KOH | 104 | −, 1.6 | 84.7 | 706 | 93 | 10000 | 17 |
| AlCu-NiCoP//AC AHS | 6 M KOH | - | 102, 1.5 | 62.8 | 750 | 93.9 | 8000 | 18 |
| NiMoS_4_-A//AC HSC | 2 M KOH | - | 101.3, 1.6 | 35 | 400 | 82 | 10000 | 19 |
| NSOH NWs//AC HSCs | 6 M KOH | 73 | −, 0–1.6 | 59.8 | 10472.3 | 91.5 | 10000 | 20 |
| 3.3.1 Metal-ion capacitor | | | | | | | | |
| NiMn-LDH@COV/CP//AC | 1 M PVA-KOH | - | 147, 1.5 | 45.93 | 752 | 85 | 5000 | 21 |
| GDPC//GDHC | 1 M NaClO_4_ | - | -, 1.5-4.2 | 156 | 355 | 73 | 10000 | 22 |
| NCS-CoSe_2_//AC | 1 M LiPF_6_ | - | -, 2.2–4.5 | 82 | 6.7 k | 88.5 | 6000 | 23 |
| Fe_3_O_4_@C//AC | LiPF_6_ | - | -, 0-4 | 110.1 | 250 | 95.7 | 1000 | 24 |
| Fe_3_O_4_/rGO//AC | 1 M LiPF_6_ | - | - | 98.8 | 343.8 | 78.9 | 1000 | 25 |
| 3.3.2 Redox-electrolyte capacitor | | | | | | | | |
| Full capacitors (AC at the positive and negative electrodes) | 10 mm TEMPO | 60 | -, 1.6 | 51 | - | 96 | 4000 | 26 |
| hrGO-2 electrodes as both anode and cathode | 1M Na_2_SO_4_  [Fe(CN)_6_]^3-/4-^ | - | 101.25, 2 | 56.25 | 35.83 k | 98 | 5000 | 27 |
| Full cell (with nanoporous carbon  Electrode) | 1 M SnF_2_\| 3 M VOSO_4_ |  | -, 1.4 | 58.4 | 3.8 k | 80 | 6500 | 28 |
| Symmetric carbon-carbon electrochemical  device | KBr | 48.8 | 92.0, 1.9 | 12.0 | - | 81 | 10000 | 29 |
| Carbon/carbon hybrid cell | 5 mol L^-1^ NaNO_3_ + 0.5 mol L^-1^ NaI | - | 60, 1.5 | 23 | 0.5 k | - | - | 30 |
| 3.3.3 Battery/capacitor | | | | | | | | |
| NiCo-LDH@PANI//AC | 6 M KOH | - | 297.8, 1.6 | 66.2 | 800 | 95.5 | 20000 | 31 |
| ZIBCHDs | ZnSO_4_ | 291.5 | -, 0.2–1.6 | 92.4 | 350 k | 97.2 | 20000 | 32 |
| Co_9_S_8_@OV-NiCo-LDH//AC | PVA/LiOH | - | -, 1.6 | 101.1 | 800 | 91.8 | 10000 | 33 |
| Mn−Ni LDOC//  AC | 1 M KOH | - | -, 2.0 | 78.2 | 499.7 | 99.3 | 5000 | 34 |
| Li_4_Ti_5_O_12_+AC/LiMn_2_O_4_+AC | 1 M LiPF_6_/ECDMC-EMC | 56.4 | -, 1.4-2.8 | 60 | 2500 | 90 | 5000 | 35 |

**References**

[1] D. Pandey, K. S. Kumar, J. Thomas *Prog. Mater. Sci.* **2024**, 141, 101219.

[2] L. J. Aaldering, C. H. Song *J. Clean. Prod.* **2019,** 241, 118343.

[3] Z. Moradi, A. Lanjan, R. Tyagi, S. Srinivasan *J. Energy Storage* **2023**, [73,](https://www.sciencedirect.com/journal/journal-of-energy-storage/vol/73/part/PC) 109048.

[4] A. Riaz, M. R. Sarker, M. H. M. Saad, R. Mohamed *Sensors* **2021**, 21, 5041.

[5] L. L. Zhang, X. S. Zhao *Chem. Soc. Rev.* **2009**, 38, 2520−2531.

[6] C. H. Ng, H. N. Lim, S. Hayase, Z. Zainal, S. Shafie, N. M. Huang *Electrochim. Acta*

**2017,** 29, 73-182.

[7] J. Zhang, J. Jiang, H. Li, X. S. Zhao *Energy Environ. Sci.* **2011**, 4, 4009-4015.

[8] R. Adalati, A. Kumar, M. Sharma, R. Chandra *Appl. Phys. Lett.* **2021***,* 118, 183901.

[9] T. Zhai, S. Xie, M. Yu, P. Fang, C. Liang, X. Lu, Y. Tong *Nano Energy* **2014**, 8, 255-263.

[10] P. Du, Y. Dong, C. Liu, W. Wei, D. Liu, P. Liu *J. Colloid Interface Sci.* **2018**, 518, 57-68.

[11] H. Zhang, F. Zhang, Y. Wei, Q. Miao, A. Li, Y. Zhao, Y. Yuan, N. Jin, G. Li *ACS Appl. Mater. Interfaces* **2021**, 13, 21217–21230.

[12] S. Jin, H. Lee, S. Yim *RSC Adv.* **2019**, 9, 31846-31852.

[13] M. Harilal, B. Vidyadharan, I. I. Misnon, G. M. Anilkumar, A. Lowe, J. Ismail, M. M. Yusoff, R. Jose *ACS Appl. Mater. Interfaces* **2017**, 9, 12, 10730–10742.

[14] L. Zhang, W. Zheng, H. Jiu, C. Ni, J. Chang, G. Qi *Electrochim. Acta* **2016**, 215, 212-222.

[15] Y. Han, Y. Lu, S. Shen, Y. Zhong, S. Liu, X. Xia, Y. Tong, X. Lu *Adv. Funct. Mater.* **2019**, 29, 1806329.

[16] H. Tian, W. Bao, Y. Jiang, L. Wang, L. Zhang, O. Sha, C. Wu, F. Gao *J. Chem. Eng.* **2018,** 354, 1132-1140.

[17] T. I. Singh, G. Rajeshkhanna, T. Kshetri, N. H. Kim, J. H. Lee *J. Mater. Chem. A* **2020**, 8, 26158-26174.

[18] L. Lyu, W. H. Antink, B.-H. Lee, C. W. Kim, E. Jung, K.-d. Seong, T. Hyeon, Y. Piao *ACS Appl. Energy Mater.* **2021**, 4, 10, 10553–10564.

[19] D. Du, R. Lan, J. Humphreys, W. Xu, K. Xie, H. Wang S. Tao *J. Electrochem. Soc.* **2017**, 164, A2881.

[20] W. Li, Z. Huang, Y. Jia, Y. Cui, P. Shi, T. Li, H. Yue, J. Wang, W. He, X. Lou *Mater. Chem. Front.* **2022**, 6, 94-102.

[21] A. Sahu, P. Pandey, S. Bhowmick, M. Qureshi *Chem. Commun.* **2023**, 59, 1038-1041.

[22] H. Liu, X. Liu, H. Wang, Y. Zheng, H. Zhang, J. Shi, W. Liu, M. Huang, J. Kan, X. Zhao, D. Li *ACS Sustain. Chem. Eng.* **2019**, 7, 12188-12199.

[23] B. Li, H. Hu, H. Hu, C. Huang, D. Kong, Y. Li, Q. Xue, Z. Yan, W. Xing, X. Gao *Electrochim. Acta* **2021,** 370, 137717.

[24] C. Han, L. Xu, H. Li, R. Shi, T. Zhang, J. Li, C.-P. Wong, F. Kang, Z. Lin, B. Li *Carbon* **2018,** 140, 296-305.

[25] J.-L. Huang, L.-Q. Fan, Y. Gu, C.-L. Geng, H. Luo, Y.-F. Huang, J.-M. Lin, J.-H. Wu *J. Alloys Compd.* **2019**, 788, 1119-1126.

[26] L. Hu, C. Shi, K. Guo, T. Zhai, H. Li, Y. Wang *Angew. Chem. Int. Ed.* **2018,** 57, 8214-8218.

[27] C. Guo, Y. Zhang, T. Zeng, D. Huang, Q. Wan, N. Yang *Carbon* **2020**, 157, 298-307.

[28] J. Lee, A. Tolosa, B. Krüner, N. Jäckel, S. Fleischmann, M. Zeiger, D. Kim V. Presser *Sustain. Energy Fuels* **2017**, 1, 299-307.

[29] Q. Li, M. Haque, V. Kuzmenko, N. Ramani, P. Lundgren, A. D. Smith, P. Enoksson *J. Power Sources* **2017**, 348, 219-228.

[30] Q. Abbas, H. Fitzek, V. Pavlenko, B. Gollas *Electrochim. Acta* **2020,** 337, 135785.

[31] H. Guo, J. Tian, M. Wang, Y. Chen, N. Wu, L. Peng, Y. Liu, X. Xu, W. Yang *New J. Chem.* **2023**, 47, 16050-16058.

[32] Z. Sun, X. Han, D. Wang *J. Energy Storage* **2023,** 62, 106857.

[33] M. Liu, L. Wang, X. Yu, H. Zhang, H. Zhang, S. Li, F. Huang *Energy* **2022**, 238, 121767.

[34] S. Jiang, Y. Qiao, T. Fu, W. Peng, T. Yu, B. Yang, R. Xia, M. Gao *ACS Appl. Mater. Interfaces* **2021**, 13, 34374–34384.

[35] D. Ruan, Y. Huang, L. Li, J. Yuan, Z. Qiao *J. Alloys Compd.* **2017**, 695, 1685-1690.
